# People See Political Opponents as More Stupid Than Evil

**DOI:** 10.1177/01461672221089451

**Published:** 2022-04-28

**Authors:** Rachel Hartman, Neil Hester, Kurt Gray

**Affiliations:** 1 The University of North Carolina at Chapel Hill, USA; 2 McGill University, Montreal, Quebec, Canada

**Keywords:** political polarization, affective polarization, meta-perceptions, social perceptions

## Abstract

Affective polarization is a rising threat to political discourse and democracy. Public figures have expressed that “conservatives think liberals are stupid, and liberals think conservatives are evil.” However, four studies (*N* = 1,660)—including a representative sample—reveal evidence that both sides view political opponents as more unintelligent than immoral. Perceiving the other side as “more stupid than evil” occurs both in general judgments (Studies 1, 3, and 4) and regarding specific issues (Study 2). Study 4 also examines “meta-perceptions” of how Democrats and Republicans disparage one another, revealing that people correctly perceive that both Democrats and Republicans see each other as more unintelligent than immoral, although they exaggerate the extent of this negativity. These studies clarify the way everyday partisans view each other, an important step in designing effective interventions to reduce political animosity.

“Conservatives think liberals are stupid, and liberals think conservatives are evil.”

—Charles Krauthammer (2002)

As Krauthammer suggests, many left-leaning Americans see conservatives as sexist, racist, xenophobic, or just plain evil (Hart, 2018; Relman, 2019); in contrast, many right-leaning Americans see liberals as irresponsible, foolish, misguided, or downright stupid (Heston, 2019; Mullaney, 2019; Rinke, 2019; Stacy, 2018). Other narratives in social and mass media paint an alternative picture. Conservatives are unsophisticated, uneducated rednecks who vote against their self-interest (Brennan, 2016; Concha, 2019; Edsall, 2018; Edwards, n.d., Gregory, 2012); liberals are callous and cruel, promoting death panels (Reynolds, 2009) and baby killings (Berrien, 2020; Hedger, 2019). These competing narratives raise a question, “What is the primary way in which liberals and conservatives disparage each other?” We present four studies examining two oft-discussed negative traits: unintelligence and immorality. Our findings demonstrate that despite common narratives pointing at differences in the way political groups see each other, in reality, both liberals and conservatives tend to view each other similarly—as more unintelligent than immoral.

## Unintelligence and Immorality

American politics has become increasingly intolerant and partisan (Brandt et al., 2014; Chambers et al., 2013; Crawford & Pilanski, 2014), driven by disagreements about policy issues (Pew Research Center, 2019a, 2019b) and by identity-based “affective polarization” (Iyengar et al., 2012, 2019; Mason, 2018). Real-world examples of affective polarization abound, from individuals refusing to help motorists who belong to the opposite party (Gutierrez, 2016; Larimer, 2016) to partisan responses to recent COVID-19 measures (Cornelson & Miloucheva, 2020; Van Bavel, 2020).

Although it is clear that liberals and conservatives dislike each other, how exactly do partisans perceive their outgroup? There has been little research addressing this question in a way that is both systematic and applicable to the real world. There is some research on the positive moral stereotypes Democrats and Republicans have of each other (Clifford, 2019). There is also some work finding that moral polarization (the distance between perceived ingroup and outgroup morality) is greater than sociability or agency polarization (Tappin & McKay, 2019), although exploratory factor analyses suggested the three domains were best conceptualized as general dislike. Although these studies are important for understanding the way partisans view each other, they do not address the question at hand, namely, how partisans view each other’s intelligence and morality.

When people say or do something “bad,” as political opponents often seem to do, two possible explanations are (a) they are unintelligent and fail to understand it was bad, or (b) they are immoral, and understood its badness but chose to do it anyway. Discussing misinformation among Rust Belt Trump supporters, author Bob O’Connor (2020) wrote, “[they] either didn’t know or didn’t care” that they believed false statements (p. 25). Journalist Chris Mooney (2007) wrote, “The Bush White House either didn’t know or didn’t care” about stem-cell research nuances, after the former president made a controversial statement on the matter. Republicans feel similarly about Democrats: they “don’t know, or don’t care, what socialism is” (Schultz, 2020); they “don’t know or don’t care that we are $16 trillion in debt and counting” (Miller, 2012); and “[Senator Bernie Sanders] either doesn’t know or doesn’t care how money, business, and the stock market work” (LeGras, 2020). These anecdotes invite a more systematic examination of partisan perceptions.

The only investigations (to our knowledge) of how political opponents viewed each other’s immorality and unintelligence were conducted by polling organizations. The Pew Research Center (2019a) found that 55% of Republicans perceive Democrats as more immoral than the average American and 47% of Democrats feel similarly about Republicans. Ratings of unintelligence were somewhat lower: 36% of Republicans viewed Democrats as more unintelligent than the average American and 38% of Democrats returned this sentiment. These data are somewhat limited by asking participants to explicitly compare their judgment with the average American—an unclear reference group, given that about half of Americans identify as the political outgroup.

Using a different approach, an Axios poll provided participants with several adjectives and asked them to select all that apply to Democrats and Republicans (Hart, 2018). Both Democrats and Republicans described the opposing group as “ignorant” more frequently than “evil.” That this alternate methodology yielded opposite results to the Pew study highlights the potential value of a systematic analysis of partisan perceptions. In our studies, we explore outgroup derogation by assessing perceptions of unintelligence and immorality with multiple items on a continuous scale and examining both perceptions of outgroups and—for comparison—ingroups.

## Theoretical Background

Although the media and polls are replete with examples of both perceived unintelligence and immorality, we draw from two bodies of literature to ground our hypothesis that perceived unintelligence will be greater than perceived immorality.

### The True Self Is Seen as Good

In the person-perception literature, researchers make the distinction between the superficial, or peripheral self, and the “true self” (for a review, see Strohminger et al., 2017). People view morality as an essential part of one’s identity, more so than personality, memory, and cognitive faculties (Strohminger & Nichols, 2014). One’s true self is who they are deep inside and changes in the true self are akin to changes in their very identity. Moreover, while people condemn and dislike others for various reasons, there is strong evidence to suggest that they still see their true self as good (Newman et al., 2015).

Perhaps surprisingly, the main finding of the true-self literature, that people see each other as fundamentally good inside, extends even to disliked and distrusted outgroups (De Freitas & Cikara, 2018). Relating directly to the present research, Cullen (2018) found that when people explain how outgroup ideology emerges, both liberals and conservatives rely more on environmental as opposed to agential explanations. This pattern of results is expected if people view their political opponents’ true selves as good—if they are doing something bad (like voting for the wrong candidate), it is not because of who they are; rather, it is because they are influenced externally.

Taken together, this line of research provides theoretical background suggesting that participants will be reluctant to view even their political and ideological opponents as immoral, and instead are likely to view them as misguided or unintelligent.

### Partisans Distrust the Other Side’s Facts

Whereas the previous section outlined a theoretical reason to expect perceptions of immorality to be lower, there is also reason to believe perceptions of unintelligence will be higher. In other words, the gap between perceived unintelligence and perceived immorality may be explained both by low levels of perceived immorality and by high levels of perceived unintelligence. One reason to expect low levels of perceived intelligence comes from the theory of naïve realism (Ross & Ward, 1997), which states that people assume that others share their perspectives because people think their perspectives are objective. If someone disagrees with you, it is because they are missing something about reality. Supporting this theory, Sammut et al. (2015) found that participants attributed more knowledge and less ignorance to ingroup members and people who shared their views than to outgroup members and people who disagreed with them. In addition, researchers investigating the use of facts versus personal experiences in cross-cutting political discussions found that political opponents tend to distrust each other’s facts (Kubin et al., 2021). Furthermore, sharing personal experiences relating to harm increased the perceived rationality of participants’ opponents, which in turn increased respect toward participants’ opponents. Thus, it is possible that animosity is at least partially driven by low levels of perceived rationality. Taken together, these studies suggest that we should expect partisans to view each other as unintelligent.

In sum, the true-self literature provides a rationale for expecting perceived immorality to be low and the naïve realism literature provides a rationale for expecting perceived unintelligence to be high. Importantly, neither of these two bodies of literature provide reason to expect asymmetric perceptions between liberals and conservatives: That is, contrary to common narratives, we have no reason to believe liberals will view conservatives as more immoral than unintelligent while conservatives will view liberals as more unintelligent then immoral (or vice versa).

## Implications

These studies shed light on partisan stereotyping, an important first step to reducing partisan animosity. If people perceive outgroups as immoral, perhaps interventions should focus on creating mutual moral perceptions (Day et al., 2014; Feinberg & Willer, 2015; Voelkel & Feinberg, 2018). If people perceive outgroups as unintelligent, perhaps a more fruitful approach would focus on encouraging views of intelligence and rationality (Kubin et al., 2021). Furthermore, if, deep down, partisans view their opponents’ true selves as good, then encouraging them to reflect on those beliefs may reduce affective polarization. Initial evidence for the benefit of reflecting on an outgroup’s true self comes from De Freitas and Cikara (2018), who found that asking participants to reflect on their beliefs about the good true self of outgroup members (in this case, Arab immigrants) reduced outgroup bias. Thus, if partisans view each other as more unintelligent than immoral, there is reason to believe that asking them to reflect on the morality of their outgroup may reduce animosity toward them.

## Current Research

Four studies—including one with a representative sample—examined perceptions of unintelligence and immorality. We used confirmatory factor analysis to test whether these are distinguishable perceptions or whether they load on a common “dislike” factor (as in Tappin & McKay, 2019). Then, we tested Krauthammer’s claim, exploring whether perceptions of unintelligence and immorality differ whether it is liberals perceiving conservatives or conservatives perceiving liberals. Do conservatives view liberals as “stupid” and liberals view conservatives as “evil”? Or do both groups see each other as more unintelligent than immoral?

In Study 1, participants rated the perceptions of ingroup and outgroup unintelligence and immorality on a 12-item scale and we tested the scale structure, investigating whether the two constructs are indeed distinct. This study provides initial evidence that both liberals and conservatives view each other as more unintelligent than immoral. Study 2 expands on the findings from Study 1 by examining perceptions of unintelligence and immorality in the context of each group’s voting patterns. Next, Study 3 further validates the findings of Studies 1 and 2 by replicating the results in a representative sample of Americans. Finally, in Study 4, we examined lay people’s meta-perceptions about how partisans view one another.

We tested the robustness of our findings across studies by making small changes to the wording of the scale and to the reference group. In Studies 1 and 2, we asked participants *how many* people in each group are unintelligent and immoral, whereas in Studies 3 and 4 we asked about *the extent to which* they are unintelligent and immoral. Furthermore, in Studies 1 and 2 we asked about “Liberals” and “Conservatives,” whereas in Studies 3 and 4 we asked about “Democrats” and “Republicans.” These changes ensured that our findings were not due to any idiosyncrasies related to wording or framing.

Data, R scripts, and Supplemental Materials for all studies are available at https://osf.io/najsf/?view_only=a13c819cf0ca40fc8cbf64c341bb0bc5. As, in all studies, our design included only one between-subject factor (political ideology), our sample sizes provided ample power to detect a medium-sized effect (e.g., Cohen’s *d* = .5; Cohen, 1988).

## Study 1: Stupid Liberals and Evil Conservatives?

This study investigates how liberals and conservatives perceive each other (and themselves) and examines two questions. First, are perceptions of unintelligence and immorality distinct? Second, how do liberals and conservatives view each other’s unintelligence and immorality? Preregistration: https://aspredicted.org/4tp8v.pdf.^1^

### Participants and Design

We recruited 531 Americans from Amazon Mechanical Turk via CloudResearch (Litman et al., 2017) for a 5-min survey (compensation: US$0.60), with 50 participants excluded for failing attention checks. The final sample was 481 (51% female, *M*_age_ = 39, 304 liberals, 177 conservatives).

### Materials and Procedure

To measure political ideology, participants indicated “if they had to choose, [whether they were] more closely aligned with liberal or conservative values” on a 7-point scale from 1 (*very liberal*) to 7 (*very conservative*).

Participants then thought about both conservatives and liberals “as a group” and indicated how many people in this group could be described with 12 negative adjectives, six related to unintelligence (not smart, irrational, not thinking clearly, illogical, don’t appreciate facts, can’t be reasoned with) and six related to immorality (not good people, immoral, have bad intentions, have bad moral character, willing to harm others, don’t care about others). Participants responded on a 1 (*almost no one*) to 7 (*almost everyone*) Likert-type scale. After completing these ratings, participants provided demographics and received debriefing.

### Results

#### Are unintelligence and immorality distinct?

To analyze whether perceived unintelligence and immorality were distinct factors, we conducted two confirmatory factor analyses, one for outgroup ratings and one for ingroup ratings. For all four studies, we performed these analyses using the “lavaan” package in R (Rosseel, 2012). See Table 1 for overall fit statistics (comparative fit index [CFI] and root mean square error of approximation [RMSEA]) and change in chi-square (Δχ^2^) between the two-factor and one-factor models. See Supplemental Materials for additional details.

**Table 1.** Fit Statistics (CFI, RMSEA, and Δχ^2^) Across Studies.

Study	Target	Factors	CFI	RMSEA	Δχ^2^
Study 1	Outgroup	Two	0.97	0.078	643.30
		One	0.86	0.175	
	Ingroup	Two	0.98	0.067	319.40
		One	0.93	0.129	
Study 2	Outgroup	Two	0.98	0.069	531.83
		One	0.83	0.177	
	Ingroup	Two	0.97	0.081	443.80
		One	0.85	0.169	
Study 3	Outgroup	Two	0.96	0.099	321.45
	Outgroup	One	0.93	0.138	
	Ingroup	Two	0.98	0.065	99.298
	Ingroup	One	0.97	0.084	
Study 4	Outgroup	Two	0.92	0.142	299.52
		One	0.80	0.226	
	Ingroup	Two	0.93	0.119	92.84
		One	0.89	0.154	
Study 4, Meta-Perceptions (ingroup)	Outgroup	Two	0.96	0.101	290.04
		One	0.82	0.201	
	Ingroup	Two	0.92	0.134	28.79
		One	0.90	0.144	
Study 4, Meta-Perceptions (outgroup)	Outgroup	Two	0.95	0.102	182.63
		One	0.85	0.171	
	Ingroup	Two	0.90	0.152	33.30
		One	0.89	0.161	

*Note.* The two-factor models fit well across all four studies, but are a much better fit than one-factor models in outgroup perceptions compared with ingroup perceptions. CFI = comparative fit index; RMSEA = root mean square error of approximation.

**Figure 1.**
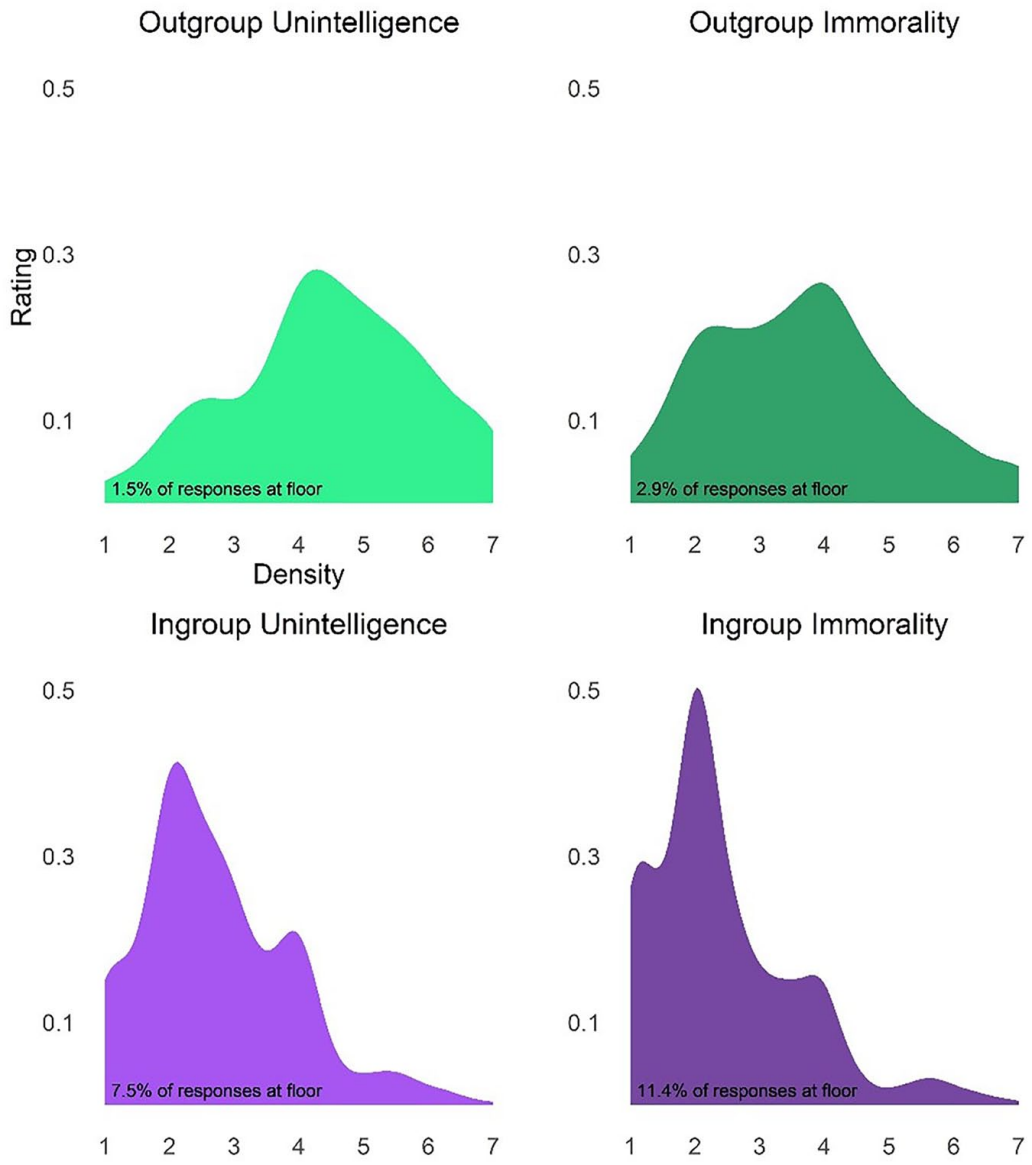
Density plot of ratings by type and target.

We first examined the distribution of ratings for each type (unintelligence and immorality) and each target group (ingroup and outgroup). As can be seen in Figure 1, outgroup ratings of both unintelligence and immorality were normally distributed, whereas ingroup ratings were skewed to the right. Despite being skewed, less than 12% of participants provided the lowest rating for ingroup unintelligence and immorality, although in some of the later studies the percentages are higher, reaching up to 36%. That we find similar interactions in the studies with more responses at floor as well as less responses at floor suggests that the interactions are not simply caused by the high percentage of responses at floor. See Supplemental Materials for distributions of the ratings in Studies 2 to 4.

**Figure 2.**
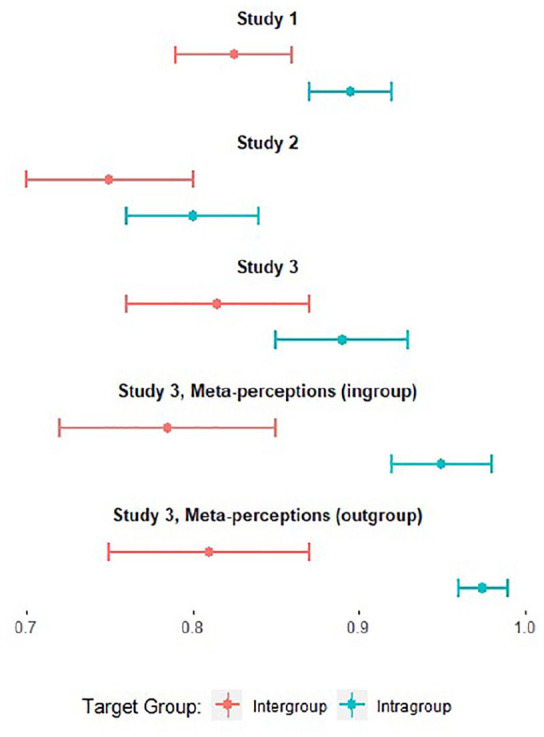
Unintelligence and immorality correlations. *Note.* In Studies 1 through 4, the correlation between ratings of unintelligence and immorality is weaker for outgroup ratings than for ingroup ratings. These results suggest that a two-factor approach has more utility for outgroup ratings than for ingroup ratings; for ingroup ratings, little additional variance in ratings is captured by the two-factor model (compared with a one-factor model in which ratings are simply “negative.”). Error bars represent 95% CIs. CI = confidence interval.

For outgroup perceptions, the two-factor model fit considerably better than the one-factor model in most of our studies (see the differences in CFI and RMSEA in Table 1). Notably, the decrement in fit between the two-factor and one-factor models is lower for ingroup ratings than for outgroup ratings, and one-factor models fit the data relatively well for ingroup perceptions. These values suggest that the two-factor model may have utility for outgroup perceptions, but not necessarily for ingroup perceptions, for which the one-factor model suffices in most cases. In Figure 2, we highlight the correlation between the two latent factors of unintelligence and immorality, which is one way to evaluate the utility or “usefulness” of the two-factor model. The higher the correlation, the less additional variance in responses is captured by modeling the data with two factors, compared with one factor. We present the correlations for all studies here. These correlations suggest that attitudes toward the outgroup meaningfully separate into “unintelligent” and “immoral” perceptions, whereas attitudes toward the ingroup are mostly captured by broadly valanced or “negative” perceptions. Although the one-factor model is sufficient for describing attitudes toward the ingroup, we nevertheless model immorality and unintelligence ratings for both outgroups and ingroups, for the sake of fitting models that match the factorial design of our studies.

#### How do liberals and conservatives disparage each other?

We used a multilevel framework to examine partisan perceptions, testing the interaction between group (ingroup vs. outgroup), rating type (unintelligence vs. immorality), political ideology (liberal vs. conservative), and ideological extremity (0–3). We used multilevel modeling to account for the fact that group and rating type were Level 1 variables (manipulated within-subjects), and political ideology and extremity were Level 2 variables (manipulated between-subjects). We started with the maximal model (i.e., full factorial) and simplified it down to two-way interactions, as no four-way or three-way interactions were significant. See the Supplemental Materials for fixed effects and for results of extremity analyses.

**Figure 3.**
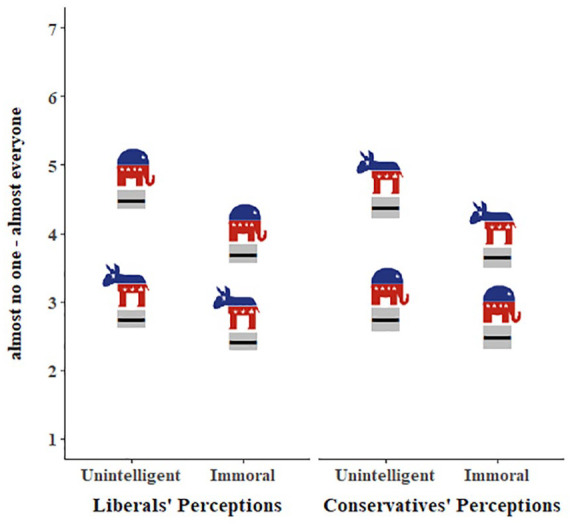
Perceived unintelligence and immorality of liberals and conservatives. *Note.* Estimated marginal means for perceptions as a function of political ideology, group, and rating type. Gray boxes represent 95% confidence intervals. Elephants and donkeys represent the groups being rated (conservatives and liberals, respectively).

There was a significant two-way interaction between target group and rating type (*B* = −.46, *SE* = .07, *p* < .001). Specifically, although ratings of unintelligence were significantly higher than ratings of immorality for both ingroups (*M*_unintelligence_ = 2.75, *M*_immorality_ = 2.46; *B* = −.33, *SE* = .07, *p* < .001) and outgroups (*M*_unintelligence_ = 4.44, *M*_immorality_ = 3.67; *B* = −.79, *SE* = .07, *p* < .001), this difference was significantly larger for outgroup ratings (ingroup *M*_diff_ = 0.29, outgroup *M*_diff_ = 0.77). There was no interaction between political ideology and rating type (*B* = .07, *SE* = .09, *p* = .41): Both liberals and conservatives viewed the opposing group as more unintelligent than immoral. See Figure 3.

### Discussion

This first study revealed that perceptions of unintelligence and immorality are distinct, at least for outgroups. Both liberals and conservatives appeared to view each other as more unintelligent than immoral—contrary to Krauthammer’s assertion. Furthermore, the difference between unintelligence and immorality was larger for outgroup versus ingroup ratings. Next, we tested whether the same patterns of perceptions would emerge for judgments surrounding a concrete issue—voting patterns.

## Study 2: Seeing Unintelligence and Immorality in People’s Voting Patterns

Study 2 examined how liberals and conservatives perceived each other after a political event. In the November elections of 2018, North Carolinians voted on six amendments to the state constitution. Three weeks after the vote, we examined how North Carolinians perceived liberals’ and conservatives’ unintelligence and immorality, after participants learned how each group voted on the amendments. Preregistration: https://aspredicted.org/we77w.pdf.

### Participants

We recruited 404 North Carolina residents on MTurk through the CloudResearch platform (Litman et al., 2017) for a 5-min survey (compensation: US$0.60), with 44 excluded for failing attention checks. The final sample was 360 (57% female, *M*_age_ = 37 years, 209 liberals, 161 conservatives) and 77% of the participants had voted in the most recent state election. We aimed to recruit 600 participants but discontinued the study after exhausting the pool of eligible participants.

### Materials and Procedure

The materials and procedure were identical to those in Study 1 apart from the amendments prompt. Participants read the six amendments (protect hunting rights, strengthen protections for crime victims, reduce the Ethics Board size, weaken the governor’s power to appoint judges, reduce the maximum state income tax, and require voter photo IDs) and learned that conservatives generally approved, and liberals generally rejected, all amendments. We asked participants why they thought so many people in each party voted the way they did (using the 12 adjectives from Study 1: six describing unintelligence and six describing immorality). Again, participants responded on a 1 (*almost no one*) to 7 (*almost everyone*) Likert-type scale. Participants then provided demographics and received debriefing.

### Results

#### How do liberals and conservatives disparage each other?

As in Study 1, we explore how liberals and conservatives view each other’s intelligence and immorality, using a multilevel model with four predictors: group (ingroup vs. outgroup), rating type (unintelligence vs. immorality), political ideology (liberal vs. conservative), and ideological extremity (0–3). We started with the maximal model (full factorial) and simplified the model down to three-way interactions, as the four-way interaction was not significant. See the Supplemental Materials for fixed effects and results of extremity analyses.

**Figure 4.**
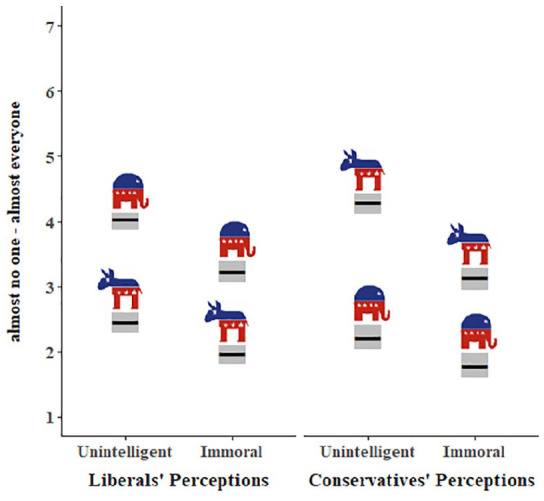
Perceived unintelligence and immorality of liberals and conservatives. *Note.* There was a marginal three-way interaction between political ideology, group, and rating type. Both liberals and conservatives view each other as more unintelligent than immoral. Gray boxes represent 95% confidence intervals.

There was a significant interaction between target group and rating type (*B* = −.50, *SE* = .11, *p* < .001). Specifically, whereas ratings of unintelligence were significantly higher than ratings of immorality for both ingroups (*M*_unintelligence_ = 2.34, *M*_immorality_ = 1.88; *B* = −.40, *SE* = .09, *p* < .001) and outgroups (*M*_unintelligence_ = 4.18, *M*_immorality_ = 3.19; *B* = −.90, *SE* = .09, *p* < .001), this difference was significantly larger for outgroup ratings (ingroup *M*_diff_ = 0.46, outgroup *M*_diff_ = 0.99). The interaction between group and rating type may be more pronounced among conservative participants; the three-way interaction involving political ideology, rating type, and group was marginally significant (*B* = −.41, *SE* = 0.22, *p* = .06). See Figure 4.

### Discussion

Like in Study 1, both liberals and conservatives rated unintelligence higher than immorality and that difference was higher for outgroup versus ingroup ratings. However, there was a marginal interaction with political affiliation: The gap between unintelligence and immorality in outgroup versus ingroup ratings was larger for conservatives than for liberals.

## Study 3: Representative Sample Replication

In Studies 1 and 2, we found that both ideological opponents and partisans view the other group as more unintelligent than immoral. Next, we sought to replicate our findings using a representative sample. We also changed the wording of the question: Instead of asking how many liberals or conservatives were unintelligent and immoral (Study 1), or why liberals or conservatives voted a certain way (Study 2), we instead asked about the extent to which participants thought the average Democrat or Republican was unintelligent or immoral. That is, we changed the targets to partisan identities instead of ideologies and changed the anchoring of the scale from *almost no one-almost everyone* to *strongly disagree-strongly agree*. Preregistration: https://aspredicted.org/as86w.pdf.

### Participants and Design

We recruited 1,350 Americans through the surveying company Lucid. Prior to answering the unintelligence and immorality measures, participants responded to a set of items for separate studies concerning attitudes toward COVID-19 restrictions. As these measures are not relevant to the present study, we do not discuss them further. The study lasted approximately 12 min and participants received compensation equivalent to roughly 10 cents per minute. We excluded 501 participants for failing any one of three attention checks and another 216 who selected “Independent” to describe their political leaning. The final sample size was 633 (67% female, *M*_age_ = 50.03, 312 Democrats and 321 Republicans).

### Materials and Procedure

To measure political identity, we asked, “How would you describe your political alignment?” Response options included “Democrat,” “Republican,” “Independent,” and “None of the above.” Respondents who selected the latter two options were excluded. Participants also indicated how they would describe their political orientation in general on a 7-point scale from 1 (*very liberal*) to 7 (*very conservative*).

After responding to the measures for the separate studies, participants responded to how they felt about Democrats and Republicans in randomized order. For each group, they indicated the extent to which they thought the provided negative adjectives (see Study 1) described the average Democrat. Participants responded on a 1 (*strongly disagree*) to 7 (*strongly agree*) Likert-type scale. After completing these ratings, participants provided demographics and received debriefing.

### Results

#### How do democrats and republicans disparage each other?

As in the previous studies, we used a multilevel framework to examine partisan perceptions, testing the interaction between group (ingroup vs. outgroup), rating type (unintelligence vs. immorality), political identity (Democrat vs. Republican), and ideological extremity (0–3, calculated from the ideology variable). Group and rating type were Level 1 variables, and political ideology and extremity were Level 2 variables. We started with the maximal model (i.e., full factorial) and simplified it down to three-way interactions, as the four-way interaction was not significant. See the Supplemental Materials for fixed effects and extremity analyses.

**Figure 5.**
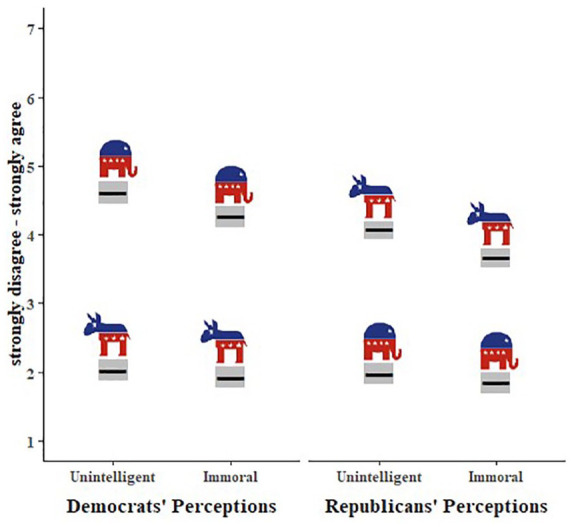
Perceived unintelligence and immorality of democrats and republicans.

The interaction between target group and rating type was significant (*B* = .26, *SE* = .09, *p* < .001). Both Democrats and Republicans rated their political ingroup similarly low on both unintelligence and immorality (*M*_unintelligence_ = 2.00, *M*_immorality_ = 1.89; *B* = −.09, *SE* = .08, *p* = .23). On the contrary, when participants rated their outgroups, they rated them as more unintelligent than immoral (*M*_unintelligence_ = 4.35, *M*_immorality_ = 3.98; *B* = −.36, *SE* = .08, *p* < .001). The ingroup difference between unintelligence and immorality (*M*_diff_ = 0.11) was significantly smaller than the outgroup difference (*M*_diff_ = 0.37). This was true for both Democrats and Republicans, although Democrats’ overall judgments of Republicans were more negative than Republicans’ judgments of Democrats (*B* = .56, *SE* = .09, *p* < .001). See Figure 5.

### Discussion

Using slightly different wording and target groups, we successfully replicated the main finding of Studies 1 and 2, namely, that both Democrats and Republicans view each other as more unintelligent than immoral.

## Study 4: Meta-Perceptions of Unintelligence and Immorality

**Figure 6.**
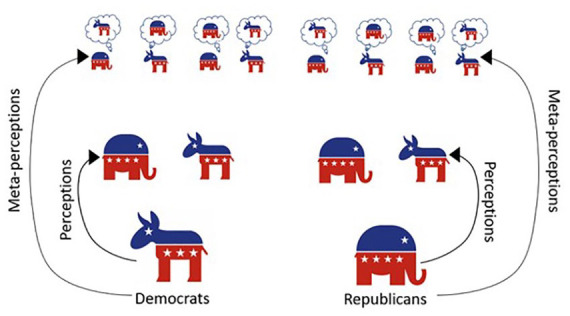
Illustration of perceptions and meta-perceptions. *Note.* Democrats and Republicans rated their ingroups’ and outgroups’ unintelligence and immorality (perceptions), as well as their meta-perceptions for how the two groups view each other and themselves.

Studies 1 to 3 provided evidence that both liberals and conservatives view each other as more unintelligent than immoral. This finding runs counter to the narratives of some public intellectuals (Epstein, 2003; Krauthammer, 2002; Muirhead, 2006), who proclaimed that conservatives view liberals as unintelligent and liberals view conservatives as immoral. This raises the question of how lay people perceive the animosity between political groups. In this final study, we examined meta-perceptions of unintelligence and immorality: What attitudes do people *think* Democrats and Republicans possess toward one another? See Figure 6 for an illustration of personal and meta-perceptions. Preregistration: https://aspredicted.org/uh2kr.pdf.

### Participants and Design

We recruited 199 Americans from MTurk via CloudResearch (Litman et al., 2017) for a 15-min survey (compensation: US$1.50), with 23 participants excluded for failing attention checks. The final sample was 176 (45% female, *M*_age_ = 36, 96 Democrats, 80 Republicans).

### Materials and Procedure

In randomized order, participants rated their meta-perceptions of (a) how their ingroup perceives their outgroup, (b) how their outgroup perceives their ingroup, (c) how their ingroup perceives their ingroup, and (d) how their outgroup perceives their outgroup. We instructed participants to rate how they thought the average Democrat perceives the average Republican (for example) regardless of their own perceptions of Republicans. Participants rated the four targets on the six unintelligence and six immorality items from the previous studies. Following each group of ratings was an attention check, asking participants which reference and target groups they had just rated.

After completing the meta-perceptions ratings, participants provided their own ratings of their ingroup’s and outgroup’s unintelligence and immorality.

Participants also responded to additional measures for a separate study and then provided their demographics and received debriefing.

### Results

#### Personal perceptions and meta-perceptions

We used a multilevel framework to examine perceptions and meta-perceptions, testing the interaction between condition (ingroup, outgroup, and the four meta-perception levels), rating type (unintelligence vs. immorality), political identity (Democrat vs. Republican), and ideological extremity (0–3). Condition and rating type were Level 1 variables, and political identity and extremity were Level 2 variables. Full results of the multilevel model are available in the Supplemental Materials.

The full factorial model showed a marginal three-way interaction between condition, rating type, and political identity, *F*(5, 2100) = 2.41, *p* = .06, as well as a significant two-way interaction between condition and political extremity, *F*(5, 2100) = 32.25, *p* < .001. For each of these interactions, we report estimated marginal means and 95% confidence intervals and consider relevant pairwise comparisons. These marginal means are estimated at the average value of all predictors not included in the marginal means.

#### How do democrats and republicans disparage each other?

**Figure 7.**
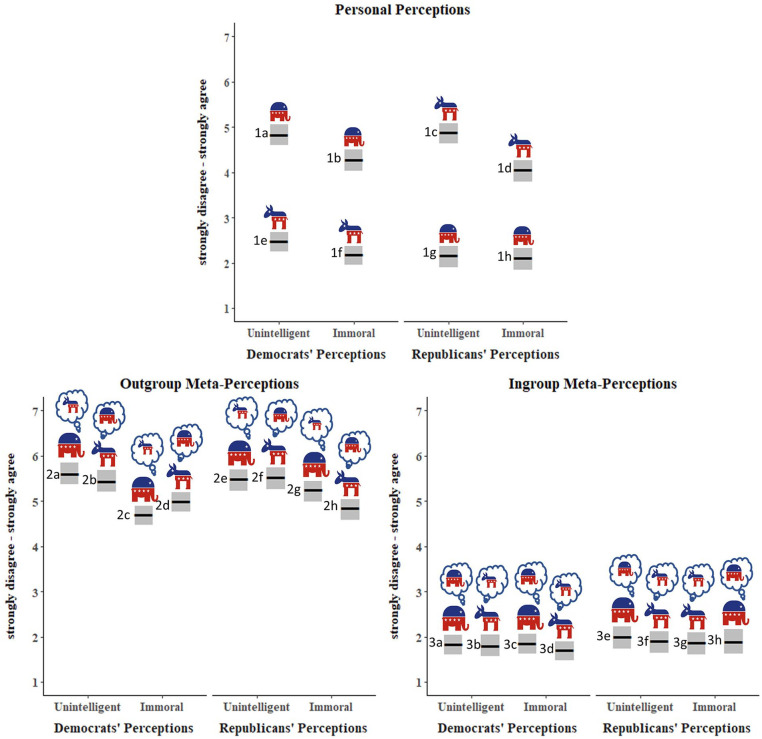
Personal perceptions and meta-perceptions of unintelligence and immorality. *Note.* Perceptions and meta-perceptions by political party, rating type, and rating target. Gray boxes represent 95% confidence intervals. Participants rated their perceptions of ingroups and outgroups (top panel) and meta-perceptions of outgroups (bottom left) and ingroups (bottom right). Elephants represent Republicans and Donkeys represent Democrats. The first bar on the bottom left (2a), for example, represents Democrat participants’ meta-perceptions of how Republicans view Democrats’ unintelligence.

The interaction between group and rating type was significant (*B* = .50, *SE* = .16, *p* < .001). Both Democrats and Republicans perceived their ingroups as similarly low in unintelligence and immorality (*M*_unintelligence_ = 2.33, *M*_immorality_ = 2.16; *B* = −.19, *SE* = .11, *p* = .37), but both viewed their outgroups as more unintelligent than immoral (*M*_unintelligence_ = 4.87, *M*_immorality_ = 4.18; *B* = −.68, *SE* = .11, *p* < .001). The ingroup difference between unintelligence and immorality (*M*_diff_ = 0.17) was significantly smaller than the outgroup difference (*M*_diff_ = 0.69) see Figure 7 (1a > 1b and 1c > 1d, respectively).

#### How do participants think democrats and republicans disparage each other?

When asked to estimate how the average Republican views the average Democrat, both Democrat and Republican participants accurately thought ratings of unintelligence would be higher than ratings of immorality (2a > 2c and 2f > 2h, respectively), but exaggerated the overall negativity, thinking Republicans view Democrats more negatively than they do (2a, c, f, h > 1c, d).

When asked to estimate how the average Democrat views the average Republican, Democrat participants accurately thought Democrats perceive Republicans as more unintelligent than immoral (2b > 2d). On the contrary, Republican participants thought Democrats perceive Republicans as equally unintelligent and immoral (2e = 2g). Again, all participants thought Democrats view Republicans more negatively than they do (2b, d, e, g > 1a, b).

There was little variability in ingroup ratings of unintelligence and immorality. As one would expect, both Democrats and Republicans viewed their ingroup as neither unintelligent nor immoral. However, perceptions of ingroups were somewhat more negative than the meta-perceptions for how groups see themselves, particularly for perceptions of the way Democrats think they view themselves (1e and 1f > 3b and 3d). See Supplemental Materials for all marginal means.

### Discussion

We found that both Democrats and Republicans see each other as more unintelligent than immoral, replicating the findings in Studies 1 and 2. We also found that their perceptions of how the political groups view each other are accurate in direction but not magnitude: Participants correctly guessed that each group views each other as more unintelligent than immoral, but thought the perceptions would be more negative than they actually are. Conversely, participants thought ingroup ratings, particularly among Democrats, would be more positive than they actually are.

## General Discussion

Our aim was twofold: to assess whether cross-party perceptions of unintelligence and immorality are distinct, and to compare liberals’ and conservatives’ perceptions of each other. Although some work suggests that dimensional complexity should be higher for ingroup members, who are often perceived as more heterogeneous (Mullen & Hu, 1989), we find greater dimensional complexity for outgroup members. Across four studies, we found that for outgroup ratings, unintelligence and immorality fell into two separate categories. On the contrary, for ingroup ratings, unintelligence and immorality were best conceived of as one factor. This might be explained by the fact that people’s experiences of negative attitudes and emotions are often more complex than their experiences of positive attitudes and emotions (Koenig-Lewis & Palmer, 2014).

Contrary to Krauthammer’s popular quote, we found that both liberals and conservatives view each other as more unintelligent than immoral. Furthermore, we found that participants accurately thought both Democrats and Republicans view each other as more unintelligent than immoral. However, participants exaggerated the magnitude of disparagement, thinking political groups have more negative views of their opponents than they actually do.

These findings replicated across four studies, when the questions were asked abstractly (Studies 1, 3, and 4) or regarding specific voting behaviors (Study 2). While preparing this manuscript, we took advantage of the Coronavirus pandemic to test whether political perceptions changed during a period of political tension. The pandemic, which began as an apolitical health threat (Holzwarth, 2020), transformed into a highly partisan issue in the United States (Newport, 2020). People on the right were eager to reopen the economy, whereas people on the left worried about the health risk (Roubein, 2020). Republicans were accused of being callous about human lives, whereas Democrats were accused of not understanding the gravity of the virus’s economic effects (Hulse, 2020). On May 14, 2020, we asked 329 MTurk workers via CloudResearch (Litman et al., 2017) to think of the way Democrats and Republicans have been reacting to the pandemic and indicate the extent to which the six unintelligence and six immoral items apply to each group. Replicating the previous four studies, both Democrats and Republicans viewed each other as more unintelligent than immoral. This timely replication provides further support for our main finding (full analyses are reported in the Supplemental Materials).

### Implications

Political polarization is at an all-time high. To bridge political divides, researchers and organizations need a solid understanding of how partisans perceive each other. The present studies show that, although liberals and conservatives often seem to disagree about moral values, the two groups still disparage each other’s intelligence more than each other’s morality. This finding replicates the results of the Axios poll (Hart, 2018), but runs counter to the Pew Research Foundation poll (Pew Research Center, 2019a). This may be a result of the question framing (i.e., “compared to the average American”). Future research should investigate the discrepant findings.

Two (competing) theories in the social psychology literature highlight the importance of morality in politics: According to Moral Foundations Theory (Graham et al., 2009; Haidt, 2012), liberals and conservatives disagree on many issues because they differ in their moral foundations. According to the Theory of Dyadic Morality (Schein & Gray, 2018), liberals and conservatives share the same moral mind, and therefore, if they come to understand this fact, they should find common ground. The findings from our studies suggest that morality is just part of the story, and perhaps not the most important part. If unintelligence, rather than immorality, drives perceptions of political groups, future research and interventions should aim to facilitate recognition of the other group’s knowledge and intelligence, rather than focus primarily on their morality.

Our fourth study found that political groups tend to overestimate the degree to which they view each other as unintelligent and immoral. This finding replicates similar findings in the literature: Partisans overestimate the extremity of positions held by each group (Ahler, 2014; Chambers et al., 2006; Lees & Cikara, 2020; Levendusky & Malhotra, 2016; Van Boven et al., 2018; Yudkin et al., 2019) and think each side dehumanizes the other more than they actually do (Moore-Berg et al., 2020). Ahler (2014) has demonstrated that alleviating misperceptions is often beneficial not only in correcting the meta-perceptions but also in mitigating the attitudes themselves. Future research should explore this method for reducing affective polarization.

### Limitations

We acknowledge several limitations to the present research. First, we restricted our data collection to American participants; thus, our findings may not necessarily generalize to partisan groups in other cultures. Second, the data we collected, to the extent they can be generalized to the American population, only reflect the participants’ perceptions of their outgroups at the time the data were collected. Notably, we collected our data prior to the storming of the Capitol on January 6, 2021. Significant events such as this one may have a large impact on political perceptions. However, the public discourse surrounding the event appeared to reflect our finding: On social and mass media, observers framed the right-wing protesters as “misled,” “brainwashed,” and “manipulated” (Hale, 2021; Kristof, 2021; Lewis, 2021).

In addition, as we only assessed perceptions of unintelligence and immorality, we do not exhaustively describe liberals’ and conservatives’ perceptions of each other. Partisans likely use many other negative adjectives to describe their opponents. Our study has high face validity, in that the focus on “stupid” and “evil” reflects cultural discourse about political groups, but it is far from a comprehensive overview of partisan disparagement. Furthermore, we acknowledge that we did not provide partisans with the opportunity to report positive views of their opponents.

Finally, there is the possibility that the general pattern of political outgroup unintelligence ratings being greater than immorality ratings is not a finding that is specific to political outgroups, but rather is a characteristic of any intergroup perceptions. Although it is certainly possible that one could observe the same pattern in other intergroup contexts, there are reasons to believe that these same patterns do not generalize across all ingroup–outgroup perceptions. For example, both men and women who endorse benevolent sexist beliefs (Glick & Fiske, 1996) are more likely to rate the gap between unintelligence and immorality to be larger for women than for men because here women are stereotyped as being both pure and in need of protection (i.e., incapable). Even in the case of other antagonistic groups, it is not necessarily the case that these same patterns occur. For example, atheists are uniquely seen by Christians as being highly immoral (but not necessarily unintelligent; Gervais, 2013; Gervais et al., 2011), whereas Christians are seen by atheists as being less competent in science, in part because of the perceived conflict between science and religion (Rios et al., 2015; Simpson & Rios, 2019).

Finally, one might argue that unintelligence perceptions were higher than immorality perceptions because of the items’ wordings. Perhaps we worded the unintelligence items more negatively, causing partisans to endorse them more. However, if this were the case, we would not have expected the same pattern of results (unintelligence > immorality) for the ingroup ratings. Another objection one might raise is that people are just averse to seeing others as evil. This may be true, but first, participants did endorse the immorality items to *some* degree, and second, we can conclude that despite the pervasiveness of political antipathy, partisans are still somewhat reluctant to view each other as immoral.

## Conclusion

As political tensions continue to rise, social and mass media are filled with narratives about why we are becoming so polarized. One often-discussed driver of polarization—and its negative impacts—is negative perceptions of the “other side.” Although it is undoubtedly true that people often see political opponents as evil, they are even more likely to see them as stupid.

## Supplemental Material

sj-docx-1-psp-10.1177_01461672221089451 – Supplemental material for People See Political Opponents as More Stupid Than Evil Click here for additional data file. Supplemental material, sj-docx-1-psp-10.1177_01461672221089451 for People See Political Opponents as More Stupid Than Evil by Rachel Hartman, Neil Hester and Kurt Gray in Personality and Social Psychology Bulletin

## Notes

1. The preregistration for Study 1 is about political context (the degree of political homogeneity in one’s social network) rather than the main hypotheses in this article; thus, our results did not match our preregistered expectations in Study 1. Based on the results of this first study, we shifted the focus of this project from political context to investigating the (a)symmetry between liberals’ and conservatives’ judgments of each other. All subsequent preregistrations concern the main hypotheses in this article.
