# Supplementary material for: People See Political Opponents as More Stupid Than Evil
Source: Pers Soc Psychol Bull. 2022 Apr 28;49(7):1014–27. doi: 10.1177/01461672221089451 (PMC10302377; doi:10.1177/01461672221089451)
Supplement: sj-docx-1-psp-10.1177_01461672221089451 – Supplemental material for People See Political Opponents as More Stupid Than Evil [file sj-docx-1-psp-10.1177_01461672221089451.docx]

# Sample Size and Demographics Across Studies

| Study | Sample size (full) | Sample size (final) | Mean Age(SD) | Gender (% male) | Ideology (% liberal/Democrat) |
| --- | --- | --- | --- | --- | --- |
| Study 1 | 531 | 481 | 39.08(12.51) | 49.7 | 63.2 |
| Study 2 | 404 | 370 | 36.9(12.30) | 41.9 | 56.5 |
| Study 3 | 1350 | 633 | 50.03(16.49) | 33.2 | 49.3 |
| Study 4 | 199 | 176 | 36.88(10.81) | 53.4 | 54.5 |
| COVID study | 413 | 329 | 37.97(12.42) | 55.0 | 50.5 |

# Confirmatory Factor Analyses

In all studies, we began by conducting two confirmatory factor analyses for our unintelligence and immorality items: one for outgroup responses and one for ingroup responses. These analyses serve two purposes: one, they measure the overall appropriateness and fit of our proposed two-factor models for ingroups and outgroups. Two, they allow us to compare the correlations between latent factors of unintelligence and immorality to test whether these variables are less strongly correlated for outgroup or ingroup ratings.

Participants responded to six unintelligence items and six immorality items. To confirm that their ratings of outgroups could indeed be separated into these two factors, we ran a confirmatory factor analysis using the “lavaan” package in R.

Across all studies, two-factor models generally fit the data better than one-factor models. However, model fit is not the only consideration for the most appropriate model; additional factors should provide significant additional information about latent factor structure. In Study 1 and Study 4 (both personal perceptions and meta-perceptions), the correlation structure and the difference in fit between the two-factor and one-factor models both suggest that, while the two-factor model is most appropriate for outgroup ratings, the one-factor model is most appropriate for ingroup ratings.

Interpreted alone, Study 2 suggests that a two-factor model for both ingroup and outgroup ratings might be most appropriate. In Study 4, the two-factor model fit only slightly better thant he one-factor model. Finally, interpreted alone, the supplementary COVID study suggests that a one-factor model for both ingroup and outgroup ratings might be most appropriate. See table below for all CFA statistics.

Table S1: confirmatory factor analyses across studies

|  |  | **Correlation between unintelligence and immorality [95% CI]** | **% shared variance** | **Item loadings** | **Chi-square**  **(all p < .001)** | **CFI** | **RMSEA [90% CI]** | **Two vs. One factor model (Chi-square difference; all p < .001)** |
| --- | --- | --- | --- | --- | --- | --- | --- | --- |
| Study 1 | Outgroup (two-factor) | .82[.79, .86] | 67 | .92-1.23 | 209.94 | .97 | .078 [.068, .090] | 643.3 |
|  | Outgroup (one-factor) |  |  | .97-1.14 | 853.29 | .86 | .175[.165, .186] |  |
|  | Ingroup (two-factor) | .89[.87, .92] | 79 | .94-1.15 | 169.01 | .98 | .067[.056, .079] | 319.4 |
|  | Ingroup (one-factor) |  |  | .97-1.11 | 488.38 | .93 | .129[.119, .140] |  |
| Study 2 | Outgroup (two-factor) | .75[.70, .80] | 56 | 1.00-1.12 | 145.99 | .98 | 0.069[.056, .082] | 531.83 |
|  | Outgroup (one-factor) |  |  | 1.00-1.26 | 677.82 | .83 | .177[.165, .189] |  |
|  | Ingroup (two-factor) | .80[.76, .84] | 64 | 1.00-1.22 | 181.43 | .97 | .081[.068, .094] | 443.8 |
|  | Ingroup (one-factor) |  |  | .81-1.16 | 625.23 | .85 | .169[.157, .181] |  |
| Study 3 | Outgroup (two-factor) | .93[.91, .94] | 86 | 1.00-1.87 | 379.81 | .96 | .099[.089, .108] | 321.45 |
|  | Outgroup (one-factor) |  |  | 1.00-1.58 | 701.26 | .93 | .138[.129, .147] |  |
|  | Ingroup (two-factor) | .96[.95, .97] | 92 | .99-1.13 | 195.15 | .98 | .065[.055, .075] | 99.29 |
|  | Ingroup (one-factor) |  |  | .99-1.10 | 294.44 | .97 | .084[.075, .093] |  |

|  | **(cont.)** | **Correlation between unintelligence and immorality [95% CI]** | **% shared variance** | **Item loadings** | **Chi-square**  **(all p < .001)** | **CFI** | **RMSEA [90% CI]** | **Two vs. One factor model (Chi-square difference; all p < .001)** |
| --- | --- | --- | --- | --- | --- | --- | --- | --- |
| Study 4 | Outgroup (two-factor) | .82[.76, .87] | 67 | .927-1.46 | 240.29 | .92 | .142[.124, .160] | 299.52 |
|  | Outgroup (one-factor) |  |  | 1.00-1.32 | 539.82 | .80 | .226[.209, .244] |  |
|  | Ingroup (two-factor) | .89[.85, .93] | 80 | .998-1.395 | 185.14 | .93 | 0.119[.101, .138] | 92.841 |
|  | Ingroup (one-factor) |  |  | .994-1.33 | 277.98 | .89 | 0.154[.136, .172] |  |
|  | Ingroup perceptions of ingroup (two-factor) | .95[.92, .98] | 90 | .962-1.339 | 221.49 | .92 | .134[.116, .153] | 28.79 |
|  | Ingroup perceptions of ingroup (one-factor) |  |  | .803-1.17 | 250.27 | .90 | .144[.126, .162] |  |
|  | Ingroup perceptions of outgroup (two-factor) | .78[.72, .85] | 61 | .966-1.12 | 147.31 | .96 | .101[.081, .120] | 290.04 |
|  | Ingroup perceptions of outgroup (one-factor) |  |  | .817-1.06 | 437.35 | .82 | .201[.184, .219] |  |
|  | Outgroup perceptions of ingroup (two-factor) | .81[.75, .87] | 66 | .857-1.05 | 150.06 | .95 | .102[.083, .121] | 182.63 |
|  | Outgroup perceptions of ingroup (one-factor) |  |  | .771-1.16 | 332.69 | .85 | .171[.154, .189] |  |
|  | Outgroup perceptions of outgroup (two-factor) | .95[.92, .97] | 90 | 1.00- 1.78 | 268.28 | .90 | .152[.134, .170] | 33.30 |
|  | Outgroup perceptions of outgroup (one-factor) |  |  | 1.00-1.74 | 301.59 | .89 | .161[.144, .179] |  |
| COVID | Outgroup (two-factor) | .92[.89, .94] | 85 | 1.00-1.12 | 155.99 | .98 | .077[.063, .091] | 266.09 |
|  | Outgroup (one-factor) |  |  | .98-1.13 | 422.08 | .93 | 0.144[.131, .157] |  |
|  | Ingroup (two-factor) | .92[.90, .94] | 85 | .92-1.14 | 209.72 | .97 | .095[.082, .108] | 194.51 |
|  | Ingroup (one-factor) |  |  | .87-1.14 | 404.23 | .93 | .140[.128, .153] |  |
|  |  |  |  |  |  |  |  |  |

# Density Plots Across Studies

Study 1


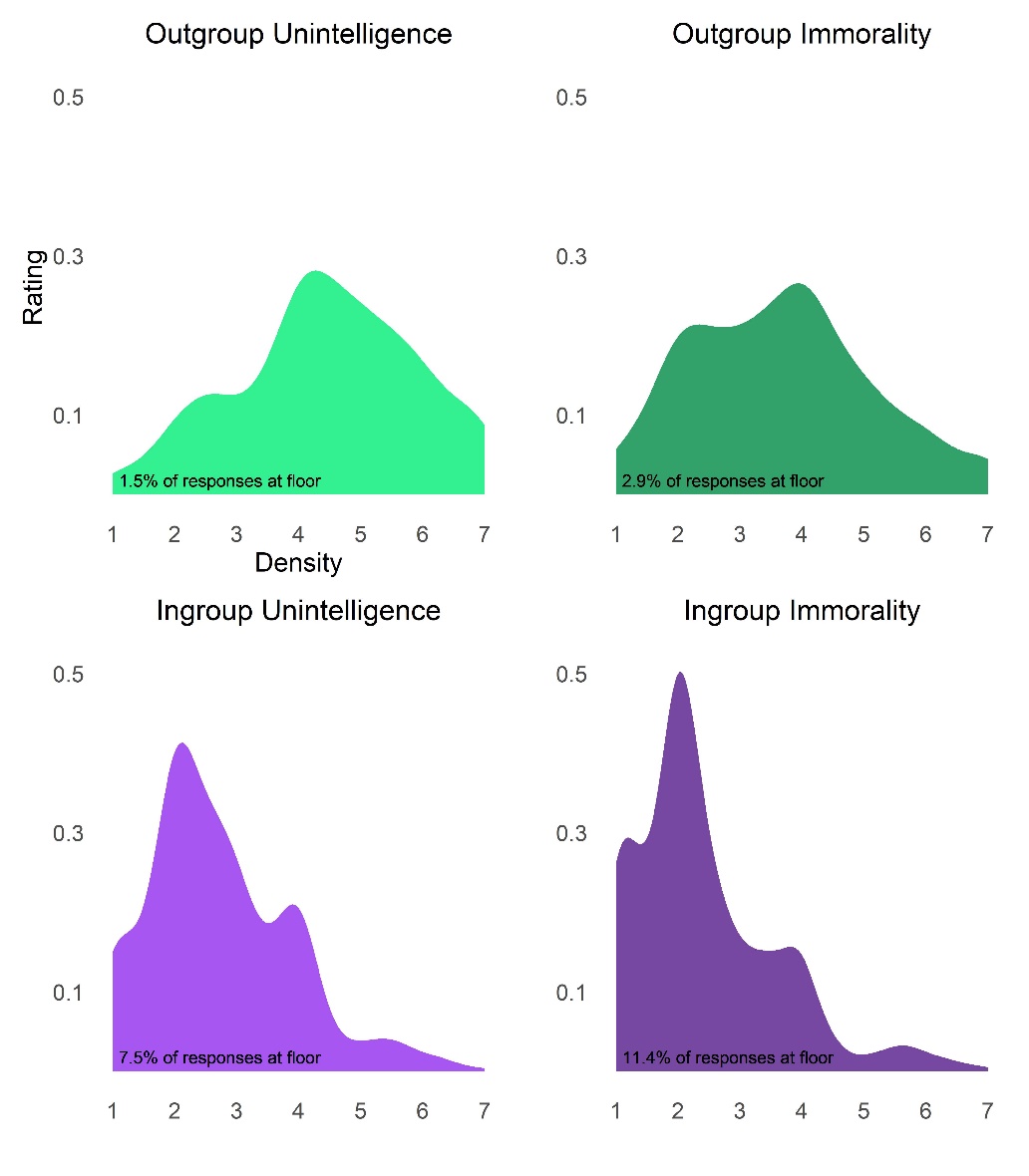


Study 2


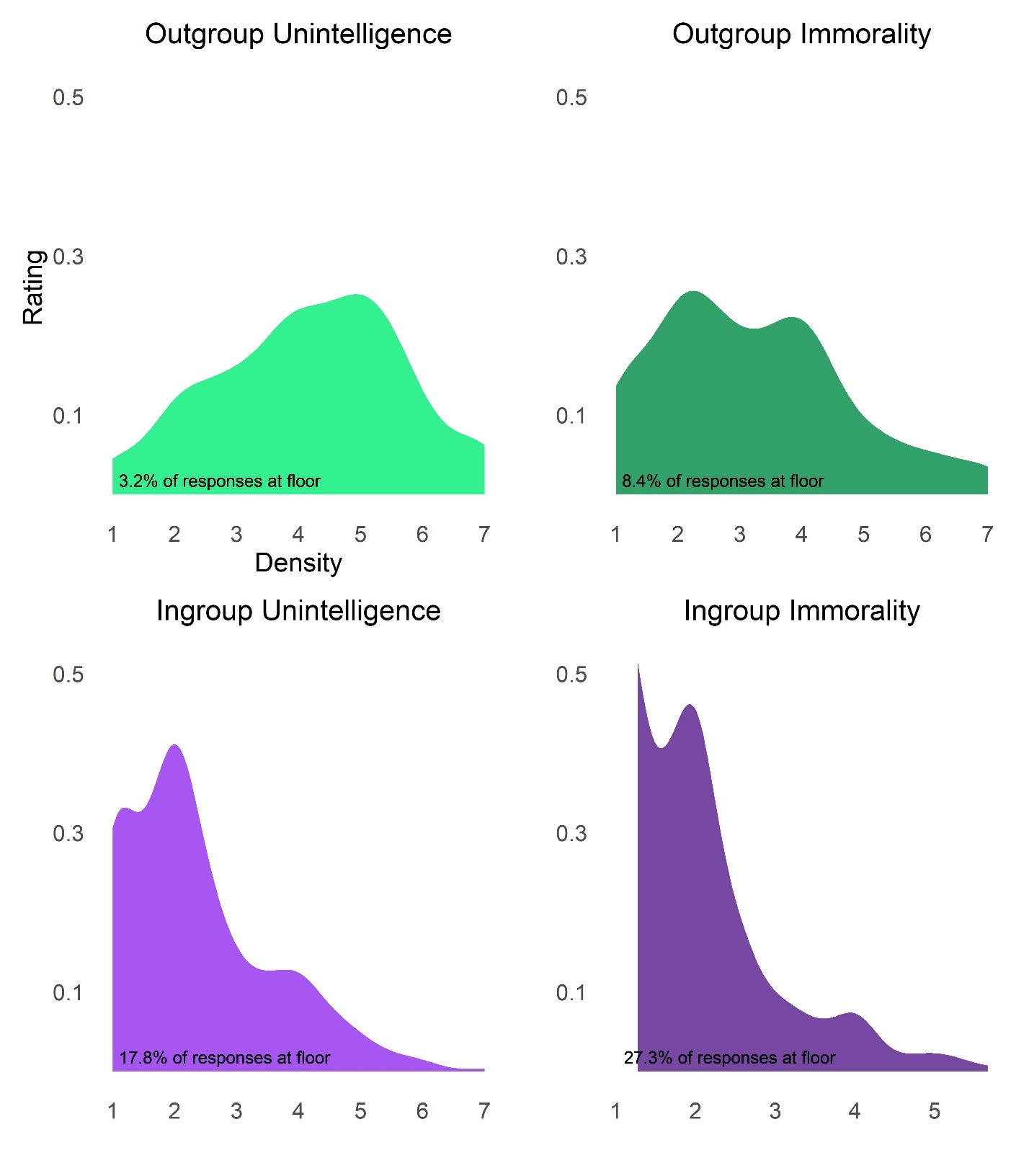


Study 3


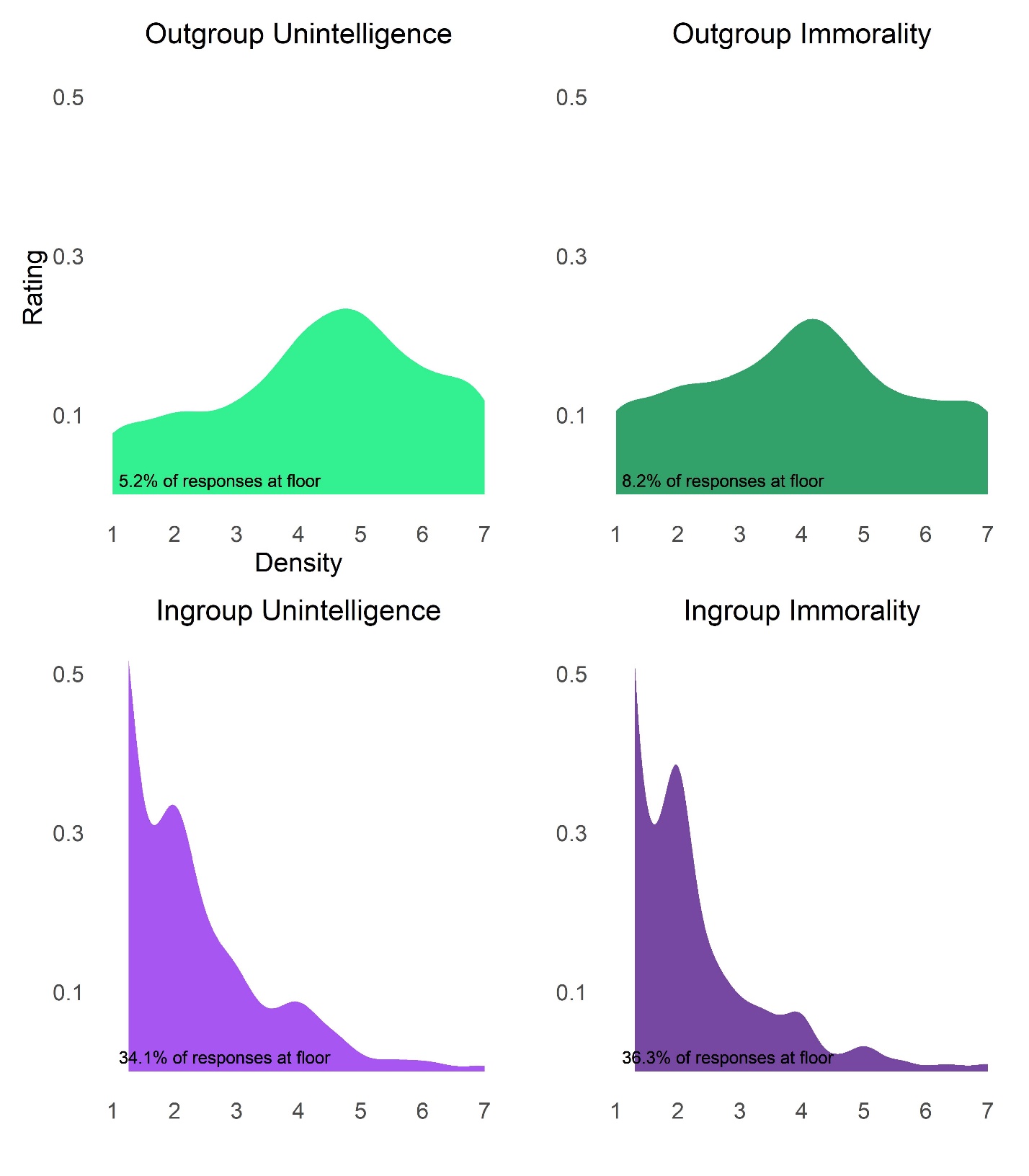


Study 4

Metaperceptions about ingroup ratings


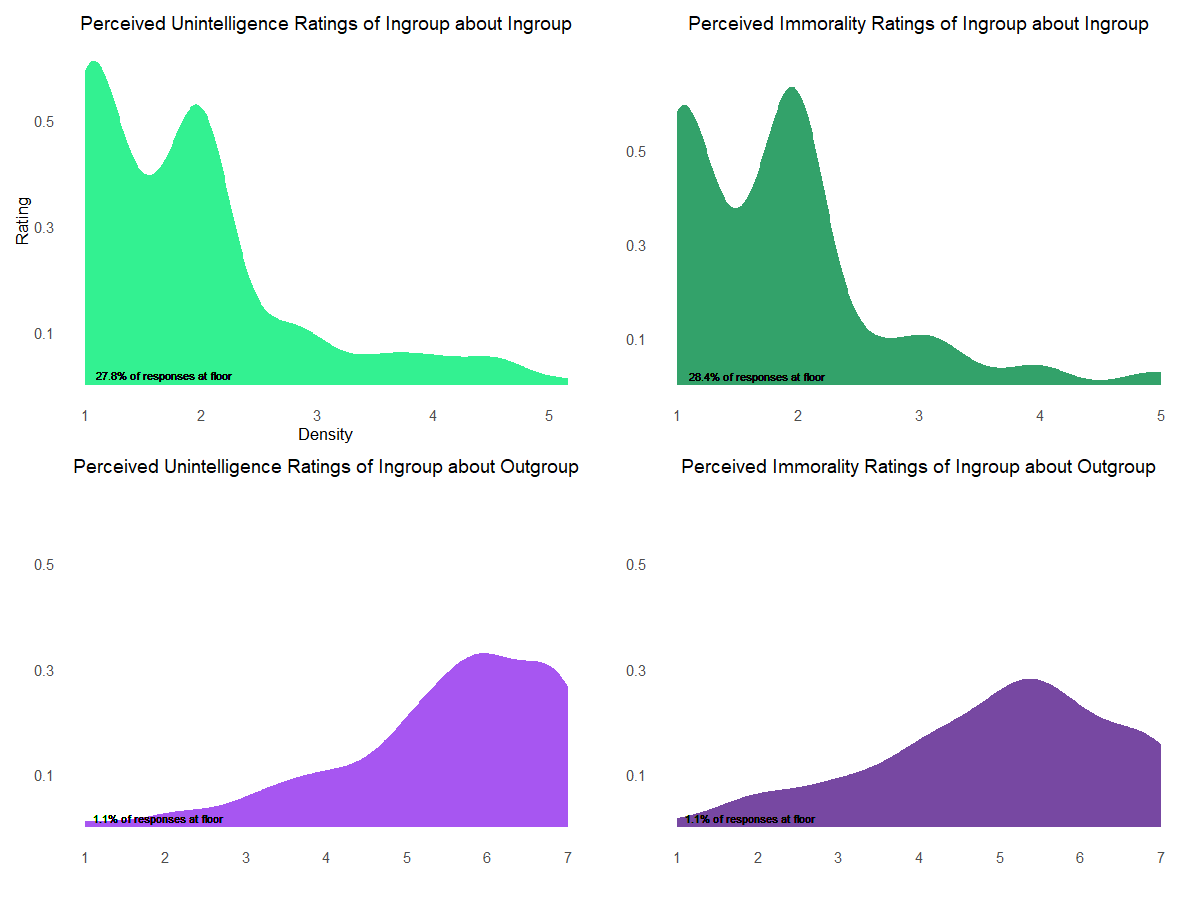


Metaperceptions about outgroup ratings


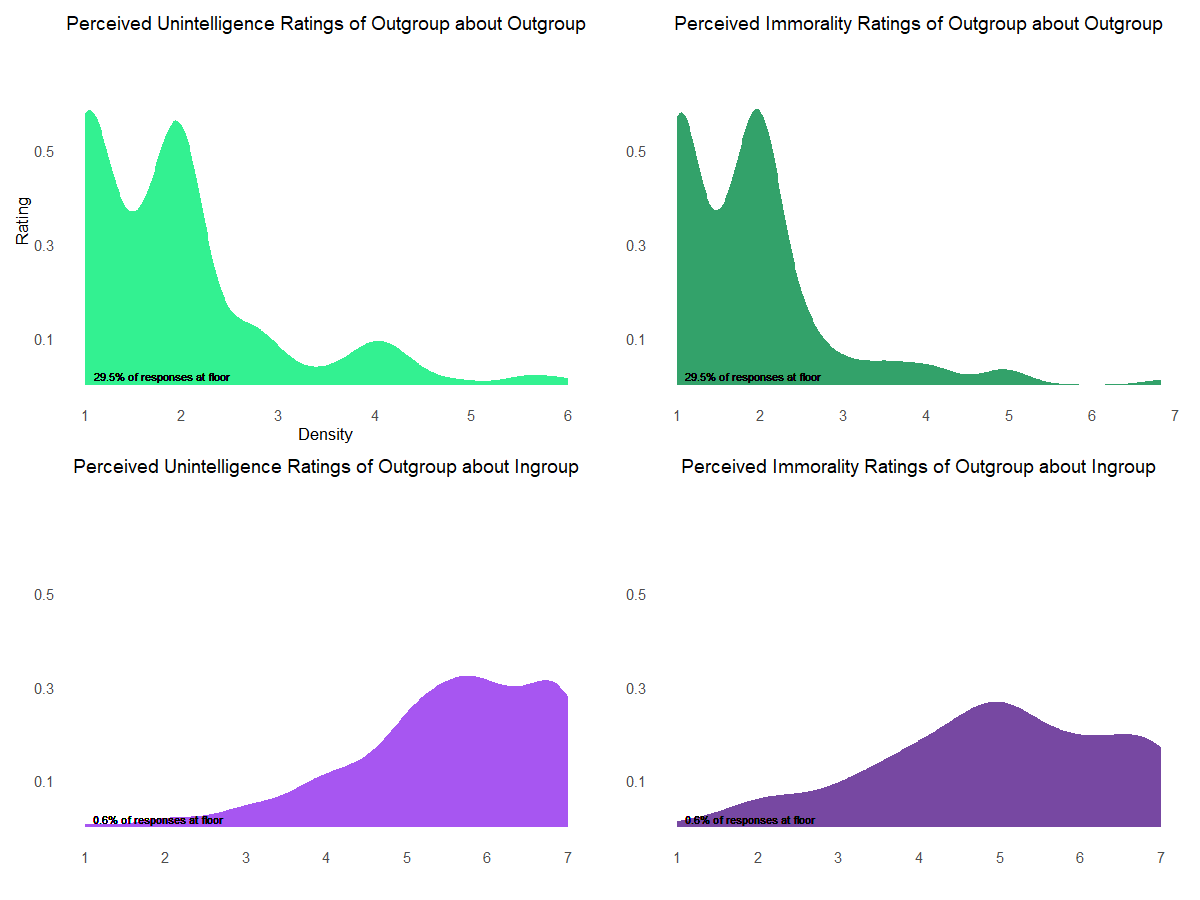


Ingroup and outgroup perceptions


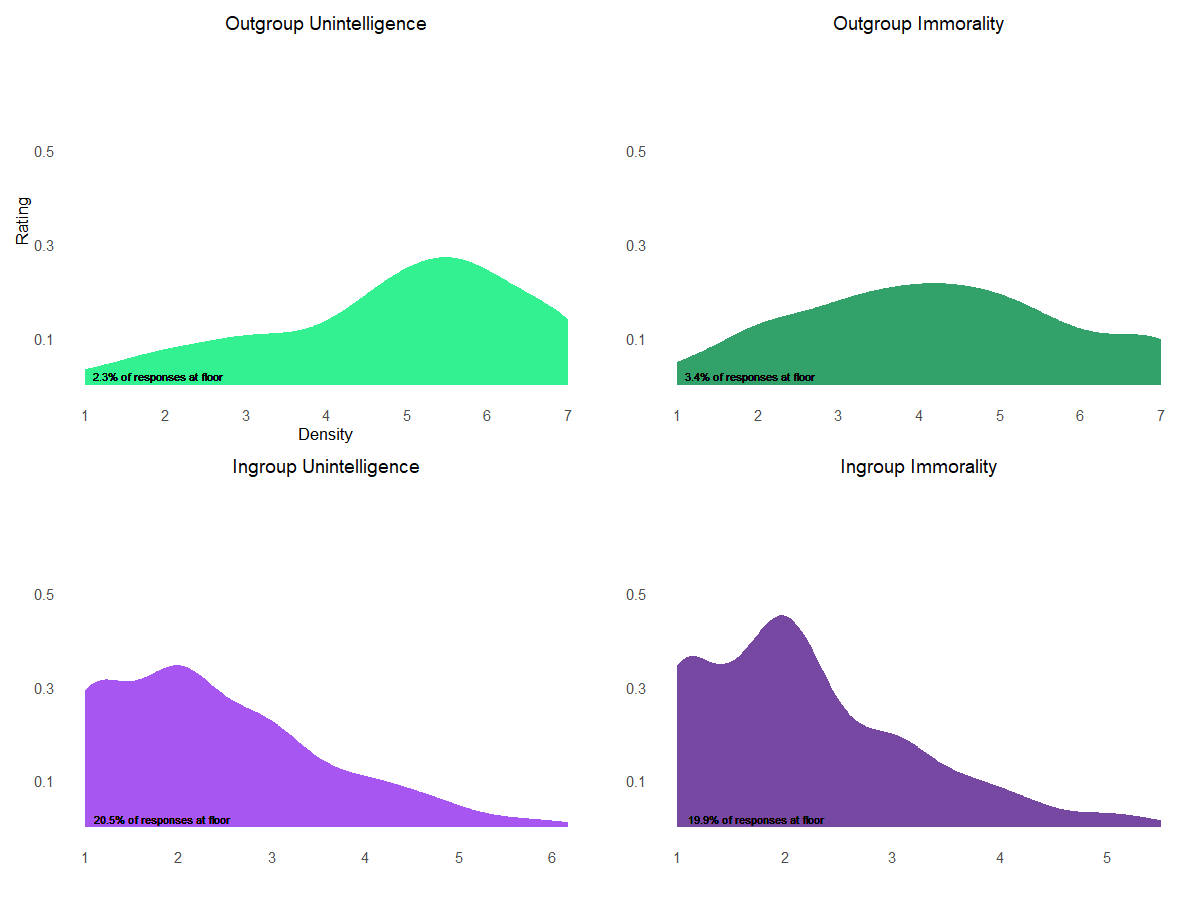


COVID study


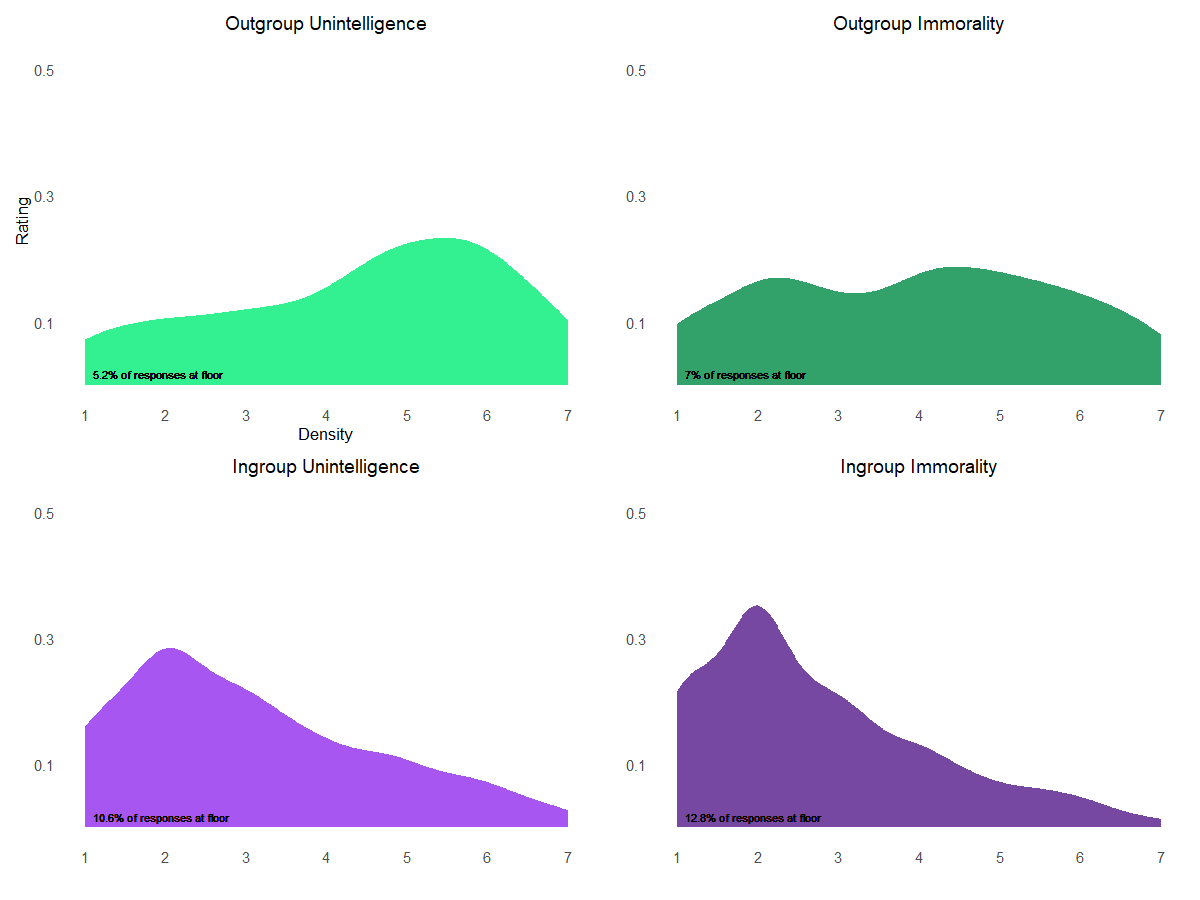


# Multilevel model results

After examining the factor structure of our unintelligence and immorality items, we created appropriate index variables for Outgroup-Unintelligence, Outgroup-Immorality, Ingroup- Unintelligence, and Ingroup- Immorality (and for study 4, meta-perception index variables for InIn, InOut, OutIn, and OutOut Unintelligence and Immorality). Then, we treated both group (Ingroup, Outgroup) and rating type (Unintelligence, Immorality) as within-subject variables. We examined these variables in a multilevel framework, such that group and rating type are Level 1 variables and political ideology and extremity are Level 2 variables.

We fit the multilevel models with four predictors: group (ingroup, outgroup), rating type (unintelligence, immorality), political affiliation (conservative or republican, liberal or democrat), and political extremity (value of 0-3). We started with the maximal model (i.e., full factorial with a four-way interaction) and simplified the model down to three-way and two-way interactions, depending on whether there were significant interactions.

In study one, there were no significant four-way or three-way interactions, so we simplified the model down to two-way interactions. See table S2 for fixed effects.

Table S2: Study 1 Fixed Effects

|  | Est. | S.E. | t val. | d.f. | p |
| --- | --- | --- | --- | --- | --- |
| Intercept | 2.75 | 0.07 | 39.04 | 1082.92 | <.001 |
| immoral1 | -0.33 | 0.07 | 4.87 | 1433.00 | <.001 |
| outgroup1 | 1.74 | 0.07 | 25.80 | 1433.00 | <.001 |
| extremity_c | -.26 | 0.07 | 3.98 | 773.73 | <.001 |
| conservative1 | 0.00 | 0.11 | 0.04 | 939.31 | 0.97 |
| immoral1:outgroup1 | -.46 | 0.08 | 5.46 | 1433.00 | <.001 |
| immoral1:extremity_c | -0.02 | 0.04 | 0.41 | 1433.00 | 0.68 |
| immoral:conservative1 | 0.07 | 0.09 | 0.82 | 1433.00 | 0.41 |
| outgroup1:conservative1 | 0.11 | 0.09 | 1.26 | 1433.00 | 0.21 |
| outgroup1:extremity_c | 0.71 | 0.04 | 15.92 | 1433.00 | <.001 |
| extremity_c:conservative1 | 0.33 | 0.10 | 3.33 | 476.00 | <.001 |

In study 2, there were no significant four-way interactions, so we simplified the model down to three-way interactions. See table S3 for fixed effects.

Table S3: Study 2 Fixed Effects

|  | Est. | S.E. | t val. | d.f. | p |
| --- | --- | --- | --- | --- | --- |
| Intercept | 2.46 | 0.09 | 28.26 | 1149.07 | <.001 |
| immoral1 | -0.49 | 0.10 | 4.75 | 1099.00 | <.001 |
| outgroup1 | 1.58 | 0.10 | 15.40 | 1099.00 | <.001 |
| conservative1 | -0.24 | 0.13 | 1.85 | 1149.64 | 0.06 |
| extremity_c | -0.48 | 0.09 | 5.42 | 1061.54 | <.001 |
| immoral1:outgroup1 | -0.33 | 0.15 | 2.24 | 1099.00 | 0.03 |
| immoral:conservative1 | 0.07 | 0.16 | 0.42 | 1099.00 | 0.67 |
| outgroup1:conservative1 | 0.51 | 0.16 | 3.24 | 1099.00 | <.001 |
| immoral1:extremity_c | 0.17 | 0.10 | 1.76 | 1099.00 | 0.08 |
| outgroup1:extremity_c | 1.02 | 0.10 | 10.69 | 1099.00 | <.001 |
| conservative1:extremity_c | 0.13 | 0.12 | 1.08 | 931.94 | 0.28 |
| Immoral1:Outgroup1:conservative1 | -0.41 | 0.22 | 1.85 | 1099.00 | 0.06 |
| Immoral1:Outgroup1:extremity_c | -0.25 | 0.11 | 2.18 | 1099.00 | 0.03 |
| Immoral1: conservative1:extremity_c | -0.10 | 0.11 | 0.86 | 1099.00 | 0.39 |
| Outgroup1: conservative1:extremity_c | -0.28 | 0.11 | 2.42 | 1099.00 | 0.02 |

In study 3, there were no significant four-way interactions, so we simplified the model down to three-way interactions. See table S4 for fixed effects.

Table S4: Study 3 Fixed Effects

|  | Est. | S.E. | t val. | d.f. | p |
| --- | --- | --- | --- | --- | --- |
| Intercept | 2.03 | 0.08 | 24.81 | 1792.88 | 0.00 |
| immoral1 | -0.10 | 0.09 | -1.14 | 1885.00 | 0.25 |
| outgroup1 | 2.59 | 0.09 | 28.12 | 1885.00 | 0.00 |
| republican1 | -0.05 | 0.11 | -0.47 | 1794.00 | 0.64 |
| extremity_c | -0.15 | 0.08 | -1.93 | 1641.02 | 0.05 |
| immoral1:outgroup1 | -0.24 | 0.13 | -1.87 | 1885.00 | 0.06 |
| immoral: republican1 | -0.02 | 0.13 | -0.16 | 1885.00 | 0.88 |
| outgroup1: republican1 | -0.47 | 0.13 | -3.67 | 1885.00 | 0.00 |
| immoral1:extremity_c | 0.05 | 0.08 | 0.63 | 1885.00 | 0.53 |
| outgroup1:extremity_c | 0.48 | 0.08 | 6.15 | 1885.00 | 0.00 |
| republican1:extremity_c | -0.12 | 0.11 | -1.08 | 1442.71 | 0.28 |
| Immoral1:Outgroup1: republican1 | -0.03 | 0.18 | -0.19 | 1885.00 | 0.85 |
| Immoral1:Outgroup1:extremity_c | -0.03 | 0.09 | -0.33 | 1885.00 | 0.74 |
| Immoral1: republican1:extremity_c | 0.03 | 0.09 | 0.29 | 1885.00 | 0.77 |
| Outgroup1: republican1:extremity_c | 0.27 | 0.09 | 2.90 | 1885.00 | 0.00 |

In study 4, there were no significant four-way interactions, so we simplified the model down to three-way interactions, of which there was one marginally significant interaction. See table S4 for fixed effects.

Table S5: Study 4 Fixed Effects

|  | Est. | S.E. | t val. | d.f. | p |
| --- | --- | --- | --- | --- | --- |
| Intercept | 1.72 | 0.13 | 13.76 | 1256.73 | <.001 |
| type_Unintelligence | 0.08 | 0.15 | 0.54 | 1886.00 | .59 |
| condition_OutIn | 2.99 | 0.15 | 19.38 | 1886.00 | <.001 |
| condition_SelfIn | 0.47 | 0.15 | 3.06 | 1886.00 | <.001 |
| condition_InOut | 3.28 | 0.15 | 21 | 1886.00 | <.001 |
| condition_OutOut | 0.14 | 0.15 | 25 | 1886.00 | .37 |
| condition_SelfOut | 2.55 | 0.15 | 0.90 | 1886.00 | <.001 |
| party_Republican | 0.17 | 0.19 | 16.53 | 1270.00 | .35 |
| extremity_Z | -0.10 | 0.12 | .88 | 1053.87 | .38 |
| type_Unintelligence: condition_OutIn | 0.82 | 0.22 | 3.77 | 1886.00 | .00 |
| type_Unintelligence: condition_SelfIn | 0.21 | 0.22 | 0.98 | 1886.00 | .33 |
| type_Unintelligence: condition_InOut | 0.37 | 0.22 | 1.69 | 1886.00 | .09 |
| type_Unintelligence: condition_OutOut | -0.10 | 0.22 | 0.46 | 1886.00 | .65 |
| type_Unintelligence: condition_SelfOut | 0.48 | 0.22 | 2.20 | 1886.00 | .03 |
| type_Unintelligence: party_Republican | -0.06 | 0.23 | 0.24 | 1886.00 | .81 |
| condition_OutIn: party_Republican | 0.37 | 0.23 | 1.61 | 1886.00 | .11 |
| condition_SelfIn: party_Republican | -0.25 | 0.23 | 1.09 | 1886.00 | .28 |
| condition_InOut: party_Republican | -0.32 | 0.23 | 1.40 | 1886.00 | .16 |
| condition_OutOut: party_Republican | -0.16 | 0.23 | 0.69 | 1886.00 | .49 |
| condition_SelfOut: party_Republican | -0.37 | 0.23 | 1.62 | 1886.00 | .11 |
| type_Unintelligence: extremity_Z | 0.01 | 0.12 | 0.04 | 1886.00 | .96 |
| condition_OutIn: extremity_Z | 0.43 | 0.14 | 3.14 | 1886.00 | <.001 |
| condition_SelfIn: extremity_Z | -0.10 | 0.14 | 0.74 | 1886.00 | .46 |
| condition_InOut: extremity_Z | 0.63 | 0.14 | 4.53 | 1886.00 | <.001 |
| condition_OutOut: extremity_Z | -0.05 | 0.14 | 0.34 | 1886.00 | .74 |
| condition_SelfOut: extremity_Z | 0.66 | 0.14 | 4.77 | 1886.00 | <.001 |
| party_Republican: extremity_Z | -0.01 | 0.15 | 0.04 | 769.28 | .97 |
| type_Unintelligence: condition_OutIn: party_Republican | -0.61 | 0.33 | 0.57 | 1886.00 | .06 |
| type_Unintelligence: condition_SelfIn: party_Republican | -0.19 | 0.33 | 0.57 | 1886.00 | .57 |
| type_Unintelligence: condition_InOut: party_Republican | 0.29 | 0.33 | 0.57 | 1886.00 | .38 |
| type_Unintelligence: condition_OutOut: party_Republican | 0.21 | 0.33 | 0.64 | 1886.00 | .52 |
| type_Unintelligence: condition_SelfOut: party_Republican | 0.32 | 0.33 | 0.97 | 1886.00 | .33 |
| type_Unintelligence: condition_OutIn: extremity_Z | -0.03 | 0.16 | 0.17 | 1886.00 | .86 |
| type_Unintelligence: condition_SelfIn: extremity_Z | -0.10 | 0.16 | 0.60 | 1886.00 | .55 |
| type_Unintelligence: condition_InOut: extremity_Z | -0.02 | 0.16 | 0.13 | 1886.00 | .89 |
| type_Unintelligence: condition_OutOut: extremity_Z | -0.01 | 0.16 | 0.06 | 1886.00 | .95 |
| type_Unintelligence: condition_SelfOut: extremity_Z | 0.02 | 0.16 | 0.12 | 1886.00 | .90 |
| type_Unintelligence: party_Republican: extremity_Z | -0.00 | 0.09 | 0.04 | 1886.00 | .97 |
| condition_OutIn: party_Republican: extremity_Z | 0.08 | 0.16 | 0.47 | 1886.00 | .64 |
| condition_SelfIn: party_Republican: extremity_Z | -0.16 | 0.16 | 0.98 | 1886.00 | .33 |
| condition_InOut: party_Republican: extremity_Z | -0.11 | 0.16 | 0.66 | 1886.00 | .51 |
| condition_OutOut: party_Republican: extremity_Z | 0.02 | 0.16 | 0.15 | 1886.00 | .88 |
| condition_SelfOut: party_Republican: extremity_Z | 0.19 | 0.16 | 1.18 | 1886.00 | .24 |

In the COVID study, there were no significant four-way or three-way interactions, so we simplified the model down to two-way interactions. See table S6 for fixed effects.

Table S6: COVID Study Fixed Effects

|  | Est. | S.E. | t val. | d.f. | p |
| --- | --- | --- | --- | --- | --- |
| Intercept | 2.62 | 0.12 | 22.09 | 991.39 | < .001 |
| immoral1 | -0.25 | 0.13 | 1.87 | 977.00 | 0.06 |
| outgroup1 | 1.84 | 0.13 | 14.00 | 977.00 | < .001 |
| extremity_c | 0.04 | 0.11 | 0.39 | 612.33 | 0.70 |
| conservative1 | 0.92 | 0.16 | 5.76 | 882.60 | < .001 |
| immoral1:outgroup1 | -0.15 | 0.15 | 0.99 | 977.00 | 0.32 |
| immoral1:extremity_c | -0.00 | 0.09 | 0.04 | 977.00 | 0.96 |
| immoral:conservative1 | -0.18 | 0.15 | 1.16 | 977.00 | 0.25 |
| outgroup1:conservative1 | -1.00 | 0.15 | 6.55 | 977.00 | < .001 |
| outgroup1:extremity_c | -0.09 | 0.09 | 1.01 | 977.00 | 0.31 |
| extremity_c:conservative1 | 0.01 | 0.14 | 0.08 | 324.00 | 0.94 |

## Main Effects

Relying on the models above, we tested main effects of group, rating type, and political ideology.

In study 1, ingroup ratings were lower than outgroup ratings, and immorality ratings were lower than unintelligence ratings. There was no main effect of political ideology. See table S7 for main effects.

Table S7: Study 1 Main Effects

|  | Estimated mean | SE | df | Lower.CL | Upper.CL | Contrast test |
| --- | --- | --- | --- | --- | --- | --- |
| Ingroup ratings | 2.60 | 0.051 | 701 | 2.50 | 2.70 | *t*(1433) = 33.522, *p* < .001 |
| Outgroup ratings | 4.06 | 0.051 | 701 | 3.96 | 4.16 |  |
| Unintelligence | 3.59 | 0.051 | 701 | 3.49 | 3.69 | *t*(1433) = 12.01, *p* < .001 |
| Immorality | 3.07 | 0.051 | 701 | 2.97 | 3.17 |  |
| Liberals’ ratings | 3.34 | 0.0557 | 476 | 3.23 | 3.45 | *t*(476) = .16, *p* = .87 |
| Conservatives’ ratings | 3.32 | 0.0734 | 476 | 3.18 | 3.47 |  |

Similarly, in study 2, ingroup ratings were lower than outgroup ratings, and immorality ratings were lower than unintelligence ratings. There was no main effect of political ideology. See table S8 for main effects.

Table S8: Study 2 Main Effects

|  | Estimated mean | SE | df | Lower.CL | Upper.CL | Contrast test |
| --- | --- | --- | --- | --- | --- | --- |
| Ingroup ratings | 2.11 | 0.053 | 654 | 2.01 | 2.22 | *t*(1099) = 28.45, *p* < .001 |
| Outgroup ratings | 3.68 | 0.0534 | 654 | 3.58 | 3.79 |  |
| Unintelligence | 3.26 | 0.0534 | 654 | 3.15 | 3.36 | *t*(1099) = 13.05, *p* < .001 |
| Immorality | 2.54 | 0.0534 | 654 | 2.43 | 2.64 |  |
| Liberals’ ratings | 2.93 | 0.0601 | 366 | 2.81 | 3.05 | *t*(366) = 0.644, *p* = .507 |
| Conservatives’ ratings | 2.87 | 0.687 | 366 | 2.73 | 3.00 |  |

Likewise, in Study 3, ingroup ratings were lower than outgroup ratings, and immorality ratings were lower than unintelligence ratings. Unlike the previous two studies, here there was a main effect of political ideology: Democrats’ overall ratings were more negative than Republicans’. See table S9 for main effects.

Table S9: Study 3 Main Effects

|  | Estimated mean | SE | df | Lower.CL | Upper.CL | Contrast test |
| --- | --- | --- | --- | --- | --- | --- |
| Ingroup ratings | 1.94 | 0.0474 | 1034 | 1.85 | 2.04 | *t*(1885) = 48.58, *p* < .001 |
| Outgroup ratings | 4.16 | 0.0474 | 1034 | 4.07 | 4.26 |  |
| Unintelligence | 3.18 | 0.0474 | 1034 | 3.08 | 3.27 | *t*(1885) = 5.37, *p* < .001 |
| Immorality | 2.93 | 0.0474 | 1034 | 2.84 | 3.02 |  |
| Democrats’ ratings | 3.21 | 0.0592 | 628 | 3.09 | 3.32 | *t*(628) = 3.731, *p* = .0002 |
| Republicans’ ratings | 2.90 | 0.0853 | 628 | 2.78 | 3.01 |  |

In study 4, ingroup ratings were lower than outgroup ratings. In table S10 we also report the contrast tests between meta-perceptions of intra and inter group ratings. In intragroup ratings, there is no difference between meta-perceptions of how one’s ingroup rates itself, and meta-perceptions of how one’s outgroup rates itself—participants expect groups to rate themselves positively. In intergroup ratings, there is also no difference between meta-perceptions of how one’s ingroup rates its outgroup, and meta-perceptions of how one’s outgroup rates its outgroup—participants expect groups to rate the opposing group negatively.

Overall, across all groups, ratings of unintelligence were higher than ratings of immorality. There was no main effect of political ideology.

Table S10: Study 3 Main Effects

|  | Estimated mean | SE | Df | Lower.CL | Upper.CL | Contrast test |
| --- | --- | --- | --- | --- | --- | --- |
| Ingroup ratings | 2.24 | 0.074 | 658 | 2.10 | 2.39 | *t*(1886) = 27.791, *p* < .001 |
| Outgroup ratings | 4.52 | 0.074 | 658 | 4.38 | 4.67 |  |
| Meta-perceptions InIn | 1.84 | 0.074 | 658 | 1.69 | 1.98 | *t*(1886) = 0.746, *p* = .976 |
| Meta-perceptions OutOut | 1.90 | 0.074 | 658 | 1.75 | 2.04 |  |
| Meta-perceptions InOut | 5.21 | 0.074 | 658 | 5.07 | 5.36 | *t*(1886) = 0.748, *p* = .976 |
| Meta-perceptions OutIn | 5.27 | 0.074 | 658 | 5.13 | 5.42 |  |
| Unintelligence | 3.67 | 0.057 | 250 | 3.56 | 3.78 | *t*(1886) = 7.443, *p* < .001 |
| Immorality | 3.32 | 0.057 | 250 | 3.21 | 3.43 |  |
| Democrats | 3.49 | 0.069 | 171 | 3.35 | 3.62 | *t*(171) = 0.241, *p* = 0.810 |
| Republicans | 3.51 | 0.076 | 171 | 3.36 | 3.33 |  |

In the COVID study, ingroup ratings were lower than outgroup ratings, and immorality ratings were lower than unintelligence ratings. Liberal ratings were also lower than conservative ratings. See table S11.

Table S11: COVID study Main Effects

|  | Estimated mean | SE | df | Lower.CL | Upper.CL | Contrast test |
| --- | --- | --- | --- | --- | --- | --- |
| Ingroup ratings | 2.91 | 0.070 | 613 | 2.78 | 3.05 | *t*(977) = 16.604, *p* < .001 |
| Outgroup ratings | 4.18 | 0.070 | 613 | 4.04 | 4.31 |  |
| Unintelligence | 3.75 | 0.070 | 613 | 3.61 | 3.89 | *t*(977) = 5.375, *p* < .001 |
| Immorality | 3.34 | 0.070 | 613 | 3.2 | 3.48 |  |
| Liberals’ ratings | 3.38 | 0.083 | 324 | 3.21 | 3.54 | *t*(324) = 2.824, *p* = .005 |
| Conservatives’ ratings | 3.71 | 0.083 | 324 | 3.55 | 3.88 |  |

## Estimated Means

In the tables below, we present the model’s estimated means for ratings of unintelligence and immorality for each group and target in each of the studies.

Table S12: Study 1 Estimated Means

| Rater and Target | Rating type | Estimate | 95% CI |
| --- | --- | --- | --- |
| Liberals rate liberals | Unintelligence | 2.75 | [2.61, 2.88] |
|  | Immorality | 2.42 | [2.28, 2.55] |
| Liberals rate conservatives | Unintelligence | 4.49 | [4.35, 4.63] |
|  | Immorality | 3.70 | [3.56, 3.84] |
| Conservatives rate conservatives | Unintelligence | 2.75 | [2.57, 2.93] |
|  | Immorality | 2.49 | [2.32, 2.67] |
| Conservatives rate liberals | Unintelligence | 4.38 | [4.21, 4.56] |
|  | Immorality | 3.67 | [3.49. 3.85] |

Table S13: Study 2 Estimated Means

| Rater and Target | Rating type | Estimate | 95% CI |
| --- | --- | --- | --- |
| Liberals rate liberals | Unintelligence | 2.46 | [2.29, 2.63] |
|  | Immorality | 1.97 | [1.80, 2.14] |
| Liberals rate conservatives | Unintelligence | 4.05 | [3.87, 4.22] |
|  | Immorality | 3.23 | [3.06, 3.40] |
| Conservatives rate conservatives | Unintelligence | 2.22 | [2.02, 2.41] |
|  | Immorality | 1.79 | [1.60, 1.99] |
| Conservatives rate liberals | Unintelligence | 4.31 | [4.11, 4.50] |
|  | Immorality | 3.15 | [2.95, 3.34] |

Table S14: Study 3 Estimated Means

| Rater and Target | Rating type | Estimate | 95% CI |
| --- | --- | --- | --- |
| Democrats rate Democrats | Unintelligence | 2.03 | [1.87, 2.19] |
|  | Immorality | 1.92 | [1.76, 2.08] |
| Democrats rate Republicans | Unintelligence | 4.62 | [4.46, 4.78] |
|  | Immorality | 4.27 | [4.11, 4.43] |
| Republicans rate Republicans | Unintelligence | 1.97 | [1.82, 2.13] |
|  | Immorality | 1.85 | [1.69, 2.01] |
| Republicans rate Democrats | Unintelligence | 4.09 | [3.93, 4.25] |
|  | Immorality | 3.68 | [3.53, 3.84] |

Table S15: Study 4 Estimated Means

| Rater and Target | Rating type | Estimate | 95% CI |
| --- | --- | --- | --- |
| Democrats rate Democrats | Unintelligence | 2.49 | [2.44, 2.74] |
|  | Immorality | 2.20 | [1.95, 2.44] |
| Democrats rate Republicans | Unintelligence | 4.84 | [4.59, 5.08] |
|  | Immorality | 4.28 | [4.03, 4.52] |
| Republicans rate Republicans | Unintelligence | 2.17 | [1.90, 2.44] |
|  | Immorality | 2.12 | [1.85, 2.39] |
| Republicans rate Democrats | Unintelligence | 4.90 | [4.63, 5.17] |
|  | Immorality | 4.07 | [3.80, 4.35] |
| Democrats’ meta-perceptions of how Democrats rate Democrats | Unintelligence | 1.81 | [1.56, 2.05] |
|  | Immorality | 1.72 | [1.48, 1.97] |
| Republicans’ meta-perceptions of how Democrats rate Democrats | Unintelligence | 5.50 | [5.23, 5.77] |
|  | Immorality | 1.88 | [1.61, 2.15] |
| Democrats’ meta-perceptions of how Democrats rate Republicans | Unintelligence | 5.45 | [5.21, 5.70] |
|  | Immorality | 5.00 | [4.76, 5.25] |
| Republicans’ meta-perceptions of how Democrats rate Republicans | Unintelligence | 5.50 | [5.23, 5.77] |
|  | Immorality | 5.26 | [4.99, 5.53] |
| Democrats’ meta-perceptions of how Republicans rate Republicans | Unintelligence | 1.85 | [1.60, 2.09] |
|  | Immorality | 1.86 | [1.62, 2.11] |
| Republicans’ meta-perceptions of how Republicans rate Republicans | Unintelligence | 1.93 | [1.65, 2.20] |
|  | Immorality | 1.90 | [1.63, 2.17] |
| Democrats’ meta-perceptions of how Republicans rate Democrats | Unintelligence | 5.61 | [5.37, 5.86] |
|  | Immorality | 4.72 | [4.47, 4.96] |
| Republicans’ meta-perceptions of how Republicans rate Democrats | Unintelligence | 5.53 | [5.26, 5.80] |
|  | Immorality | 4.85 | [4.58, 5.13] |

Table S16: COVID Study Estimated Means

| Rater and Target | Rating type | Estimate | 95% CI |
| --- | --- | --- | --- |
| Liberals rate liberals | Unintelligence | 2.62 | [2.39, 2.85] |
|  | Immorality | 2.38 | [2.14, 2.61] |
| Liberals rate conservatives | Unintelligence | 4.46 | [4.22, 4.69] |
|  | Immorality | 4.06 | [3.83, 4.29] |
| Conservatives rate conservatives | Unintelligence | 3.54 | [3.31, 3.78] |
|  | Immorality | 3.12 | [2.88, 3.35] |
| Conservatives rate liberals | Unintelligence | 4.38 | [4.15, 4.62] |
|  | Immorality | 3.81 | [3.57, 4.04] |

## Extremity

Study 1

Unsurprisingly, there was a group by extremity interaction (B = .71, SE = .03, p < .001): participants with more extreme political beliefs tended to rate the ingroup more positively and the outgroup more negatively. However, participants’ ideological extremity did not interact with rating type: both moderates and extremists view the outgroup as more unintelligent than immoral (B = -.02, SE = .04, p = .68). We did find an unexpected extremity by affiliation interaction, which we graphed separately for ingroups and outgroups for ease of interpretation. See figure S1 below. For ingroup ratings, extremity plays a larger role among liberals: liberal ideologues rate the ingroup much more positively than moderates (upper left), whereas conservatives rate their ingroup similarly across all levels of extremity (upper right). When it comes to outgroup perceptions, the reverse is true. While ratings do become more negative for both liberals and conservatives as extremity increases, they increase at a slower rate for liberals (lower left) than conservatives (lower right).


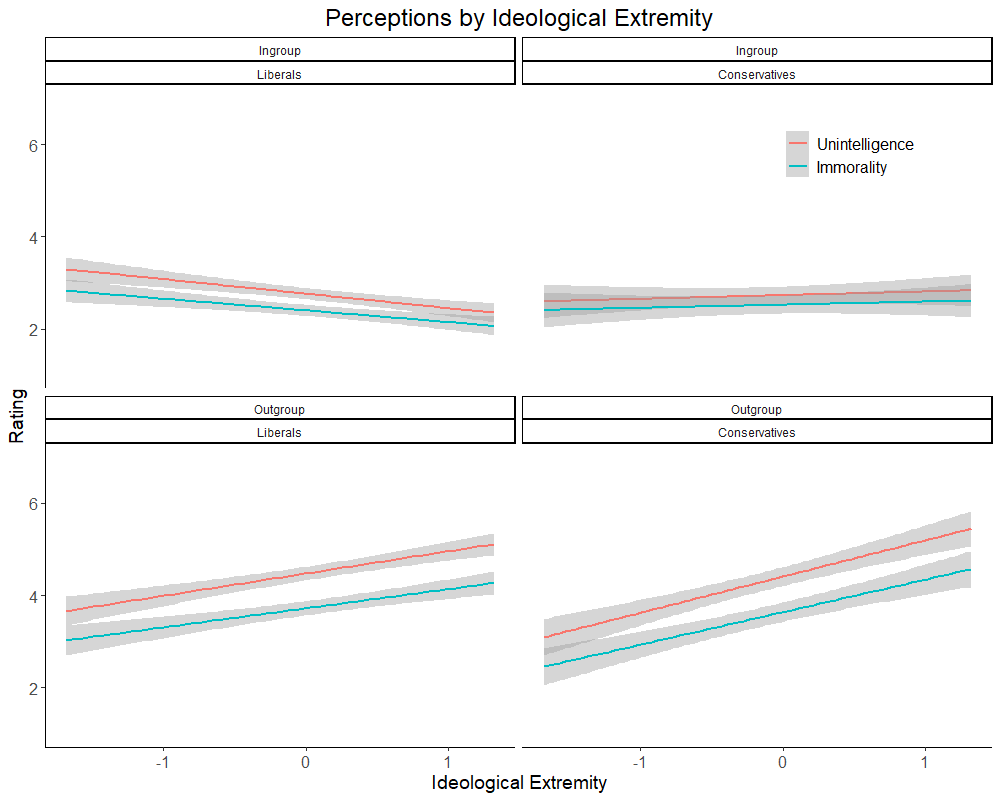


Figure S1: Study 1 Ideological Extremity

Study 2

The interaction between rating type, political ideology, and extremity was not significant (B = -.10, SE = 0.11, p = .39). In other words, ideological extremity did not alter the relationship between political ideology and rating type: liberals and conservatives both view each other as more unintelligent than immoral, regardless of how closely they identify with their politics. We did observe a Group x Rating Type x Extremity interaction. See figure S2 . For ingroup ratings (top half of the panel), as participants’ ideological extremity increased, the difference between unintelligence and immorality ratings became smaller. On the other hand, for outgroup ratings (bottom half of the panel), as extremity increased so did the gap between unintelligence and immorality ratings. However, neither of these two way interactions was significant (p = .08 and p = .40, respectively). In other words, the 3-way interaction appears to be capturing a difference in the trends, although these trends are not statistically significant in and of themselves. This pattern was the same for liberals and conservatives.


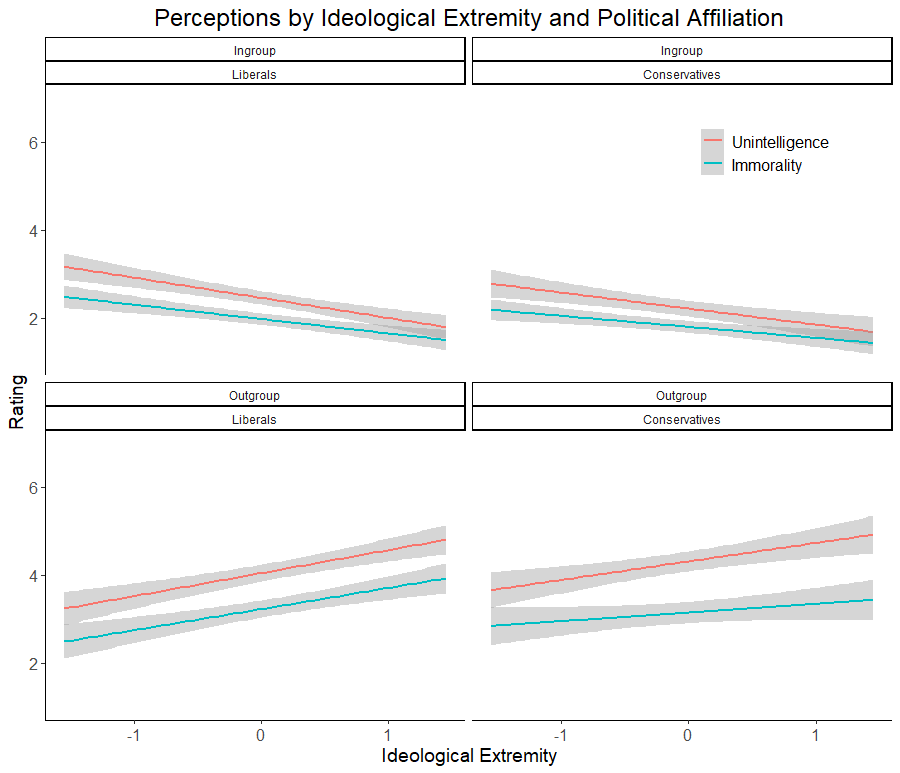


Figure S2: Study 2 Ideological Extremity

Next, we observed an Extremity x Group x Political Affiliation interaction, see figure S2. Collapsing across rating type, extremity moderated ingroup ratings similarly for liberals (top left) and conservatives (top right), but had a slightly stronger effect on outgroup ratings for liberals (bottom left) vs. conservatives (bottom right). That is, the difference between moderate and extreme liberals’ ratings of conservatives is larger than the difference between moderate and extreme conservatives’ ratings of liberals.

Study 3

There was a significant group x party x extremity interaction (B = .27, SE = .09, p < .001). When rating the ingroup, there was no interaction between party and extremity. The more closely participants identified with their ingroup the less negatively they rated their ingroup, and this was true for both Democrats and Republicans. However, when rating the outgroup, the relationship between extremity and rating depended on the participants affiliation, such that Republican moderates rated outgroups less negatively than Democrat moderates rated outgroups. As in the previous studies, participants’ ideological extremity did not interact with rating type: both moderates and extremists view the outgroup as more unintelligent than immoral (B = -.03, SE = .18, p = .85).

See figure S3


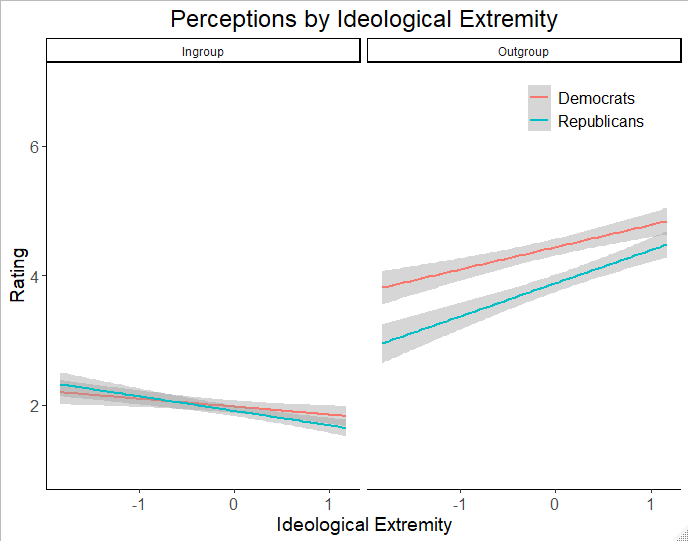


Figure S3: Study 3 Ideological Extremity

Study 4

The interaction between rating type, ideology, and extremity was not significant (B = -.00, SE = 0.16, p = .96). In other words, as in studies 1 and 2, ideological extremity does not alter the relationship between political ideology and rating type: liberals and conservatives both view each other as more unintelligent than immoral, regardless of their ideological extremity.

When outgroup members are the target of evaluation, negative ratings increase as extremity increases for actual perceptions, B = .65, 95% CI [.50, .80]; meta-perceptions for how the ingroup perceives the outgroup, B = .37, 95% CI [.23, .52]; and meta-perceptions for how the outgroup perceives the ingroup, B = .50, 95% CI [.35, .65]. In other words, people whose own political beliefs are more extreme not only hold more negative attitudes toward the outgroup, but also believe that both political ingroups and outgroups also hold more negative attitudes toward the opposing political group.

When ingroup members are the target of evaluation, participants’ own negative ratings decrease as extremity increases, B = ­­­­‑.19, 95% CI [‑.34, ‑.04]. However, meta-perceptions do not vary by extremity, Bs = .02 and .00. These results suggest an asymmetry in how extremity influences meta-perceptions: people with more extreme political beliefs perceive greater outgroup negativity, but not greater ingroup positivity. See Figure S4.


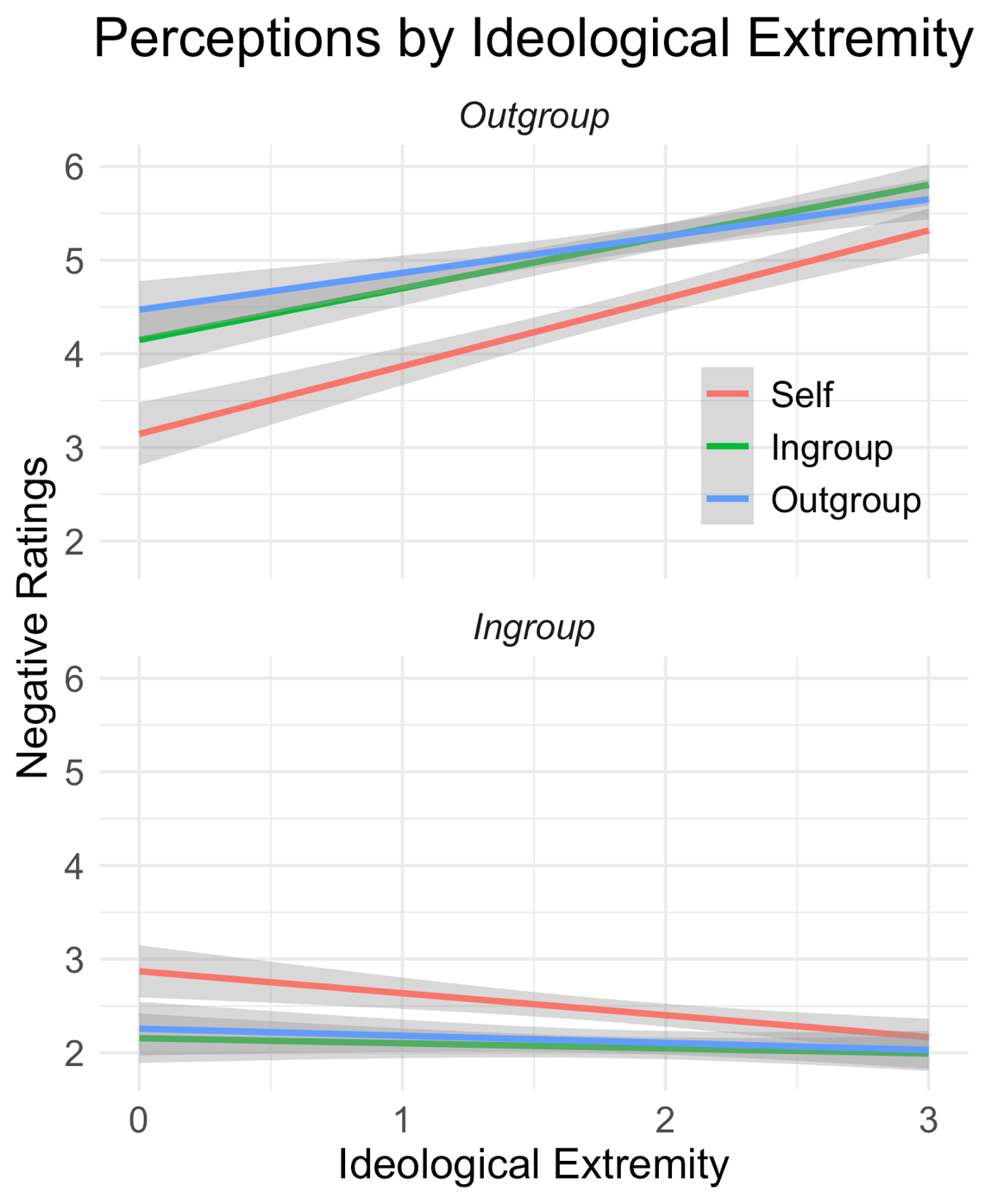


Figure S4: Study 4 Ideological Extremity

COVID study

Unlike the other three studies, there were no significant effects of ideological extremity, nor was it involved in any interactions.
